# A xeno-free human iPSC-derived prostate organoid platform for multilineage differentiation and genetic manipulation

**DOI:** 10.1016/j.crmeth.2026.101538

**Published:** 2026-07-29

**Authors:** Neha Shaikh, Matthew Teasdale, Lewis J. Walker, Laura Wilson, Rachel Howarth, Sara Saleem, Jamie Logsdon, Anastasia C. Hepburn, Ryan Nelson, Qiuyu Lian, Aleyda C. Elizondo, Rafiqul Hussain, Jonathan Coxhead, Luke Gaughan, Majlinda Lako, Emma Scott, Craig N. Robson, Ben Simons, Simon W. Hayward, Douglas W. Strand, Rakesh Heer, Adriana Buskin

**Affiliations:** 1 Newcastle University, Newcastle, UK; 2 University of Cambridge, Cambridge, UK; 3 Endeavor Health Research Institute, Evanston, IL, USA; 4 Pritzker School of Medicine, University of Chicago, Chicago, IL, USA; 5 UT Southwestern Medical Center, Dallas, TX, USA; 6 Imperial College London, London, UK

**Keywords:** stem cells, organoid model, prostate development, epithelial-stromal interactions, androgen receptor signaling, lineage specification, tissue differentiation, single-cell transcriptomics, xeno-free culture, disease modeling

## Abstract

Current prostate organoid models rely on tissue-derived material or animal components and lack epithelial and stromal complexity. We defined a xeno-free system to generate human prostate organoids from induced pluripotent stem cells with consistent multilineage differentiation. Organoids formed as free-floating 3D aggregates self-organized into the epithelial and stromal domains with basal, luminal, neuroendocrine, fibroblast, and smooth muscle markers. In an alternative modular co-culture system, engineered epithelial progenitors aggregated with wild-type mesenchymal progenitors, enabling compartment-specific manipulation. Androgen receptor (AR)-overexpressing organoids showed increased epithelial AR and prostate-specific antigen (PSA) expression and proliferation. Single-cell transcriptomics, together with qPCR and immunostaining, confirmed prostate lineage specification and tissue organization. This xeno-free platform provides a reproducible, scalable, and genetically tractable model to study *in vitro* prostate lineage programs, epithelial and stromal interactions, and disease biology.

## Highlights

• Generation of xeno-free human prostate organoids from human iPSCs
• Multilineage differentiation of epithelial and stromal prostate compartments
• Androgen receptor signaling promotes epithelial differentiation in organoids
• Compartment-specific engineering of epithelial lineages in prostate organoids

## Motivation

Current prostate organoid systems rely heavily on tissue-derived material, animal-derived matrices, or rodent stromal support, limiting reproducibility, scalability, and experimental manipulation. Human iPSC-derived systems offer an alternative approach, although fully defined xeno-free conditions and consistent multilineage organization remain challenging to achieve. Here, we developed a xeno-free and scaffold-free prostate organoid platform that supports epithelial and stromal differentiation from human iPSCs, while also enabling compartment-specific genetic manipulation within the epithelial lineage. The system provides a reproducible human model to study prostate development, epithelial-stromal interactions, and disease biology under defined conditions.

## Graphical abstract

**Figure:**
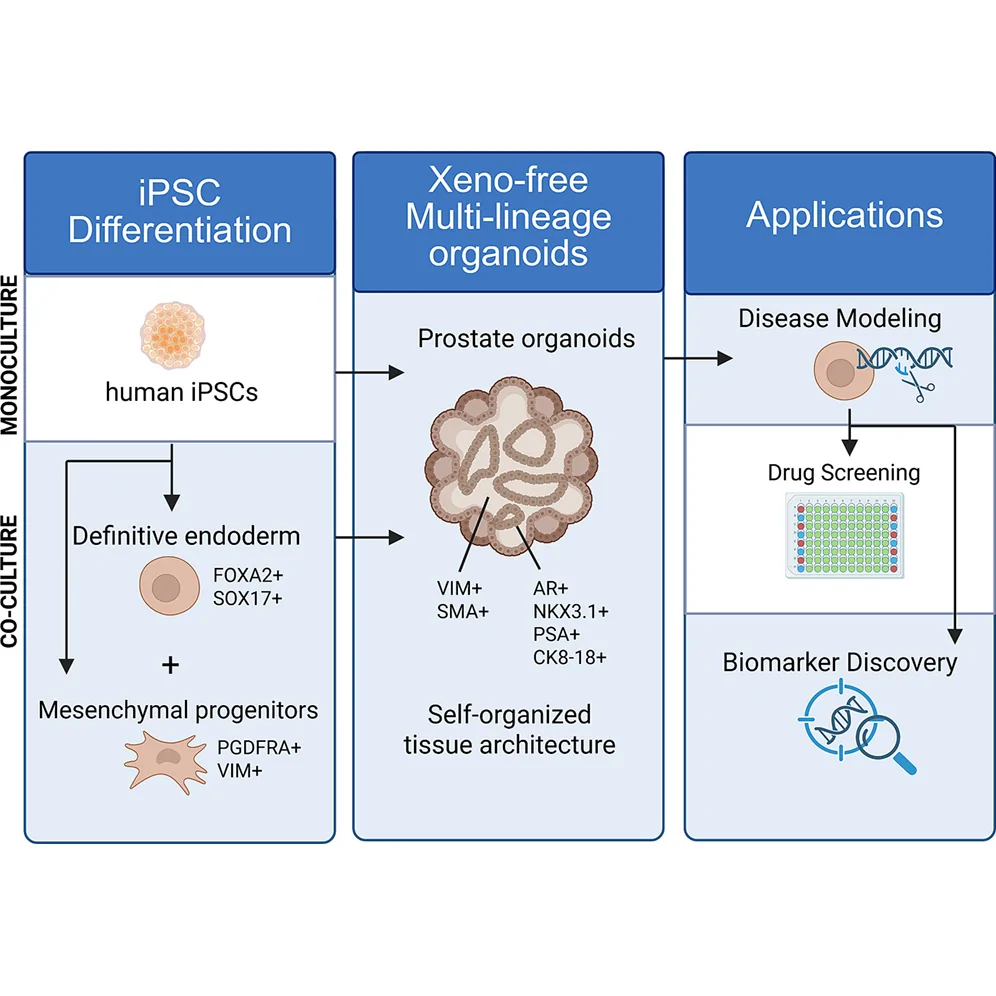

## Teaser

Shaikh et al. develop a defined, xeno-free, scaffold-free human iPSC-derived prostate organoid platform supporting epithelial and stromal differentiation. Organoids generated through monoculture or modular co-culture approaches enable compartment-specific genetic manipulation and recapitulate key features of prostate tissue organization, providing a reproducible platform for developmental and disease studies.

## Introduction

Prostate diseases affect hundreds of millions of men globally, with benign prostatic hyperplasia (BPH), prostate cancer, prostatitis, and lower urinary tract symptoms imposing a substantial and growing health burden.^1^^,^^2^ Although an area of intense research, progress is limited by the scarcity of physiologically relevant, human-specific models that accurately reflect the complexity of tissue histology and cellular differentiation. For example, prostate cancer researchers rely on fewer than ten well-characterized prostate cancer cell lines. While these lines have contributed substantially to our understanding of androgen receptor (AR) signal targeting as a central approach to treating advanced prostate cancer, they are inherently limited by their 2D structure and the absence of tumor heterogeneity and stromal-epithelial interactions.^3^

Tumor slice cultures offer the advantage of preserving native tissue architecture, but they are short-lived, difficult to scale, and exhibit transcriptomic stress responses that cloud experimental analysis.^4^ Genetically engineered mouse models (GEMMs) and patient-derived xenografts (PDXs) remain invaluable for *in vivo* validation, yet both differ from human prostate tissue in stromal composition, AR signaling, and treatment response.^5^^,^^6^^,^^7^^,^^8^ GEMMs reflect mouse-specific biology that differs from human prostate tissue. PDX models, in contrast, retain some human biology, although the stromal environment changes with the increasing passage number, and grafts require either immunodeficient or humanized hosts with no or limited immune responses. Animal-based models face constraints related to cost, engraftment efficiency, and long-term stability.^9^^,^^10^^,^^11^

Patient-derived organoids (PDOs) have significantly advanced the field by preserving patient-specific genomic and phenotypic features.^12^^,^^13^^,^^14^ However, PDO systems remain limited by their reliance on advanced-stage or metastatic samples, inefficiency in culturing treatment-naive tumors, and poor preservation of the stromal compartment.^15^^,^^16^^,^^17^ While 3D organoid technology has transformed disease modeling in other epithelial tissues, robust, isogenic, scalable, and experimentally tractable models to guide personalized therapy in prostate cancer are still lacking.^18^ Recent studies have begun to address these limitations by incorporating stromal components into culture systems and by generating human organoids from the differentiation of pluripotent stem cells as an alternative to tissue-derived material.^19^^,^^20^

Tissue recombination studies have shown that rodent urogenital mesenchyme (UGM) can direct the differentiation of human prostate epithelial cells into organized, functional prostate tissue *in vivo*. These models have demonstrated that stromal-epithelial interactions are essential for prostate organogenesis, supporting the development of basal and luminal epithelial lineages within a mesenchymal-derived stromal compartment formed by the inductive rodent UGM.^21^^,^^22^ Building on these principles, we previously demonstrated that human induced pluripotent stem cells (iPSCs) can be directed toward prostate epithelial fate through co-culture with rat UGM, generating prostate tissue both *in vivo* and *in vitro* that included neuroendocrine cells and a supporting stromal niche, thereby reproducing multiple cell types found in human prostate tissue.^23^ While this established the developmental potential of iPSCs for prostate modeling, the use of animal-derived components such as UGM and Matrigel limits reproducibility, scalability, affordability, and translational applicability.

In the present study, we present a defined, xeno-free protocol for generating human prostate organoids from iPSCs. These animal-free approaches yield self-organizing structures that capture key aspects of prostate lineage specification and tissue organization, with epithelial and stromal compartments arranged in distinct domains. The platform is reproducible and scalable and supports studies of differentiation, stromal-epithelial interaction, lineage specification, and disease modeling.

## Results

### Prostate organoids self-organize and recapitulate prostate architecture

**Figure 1:**
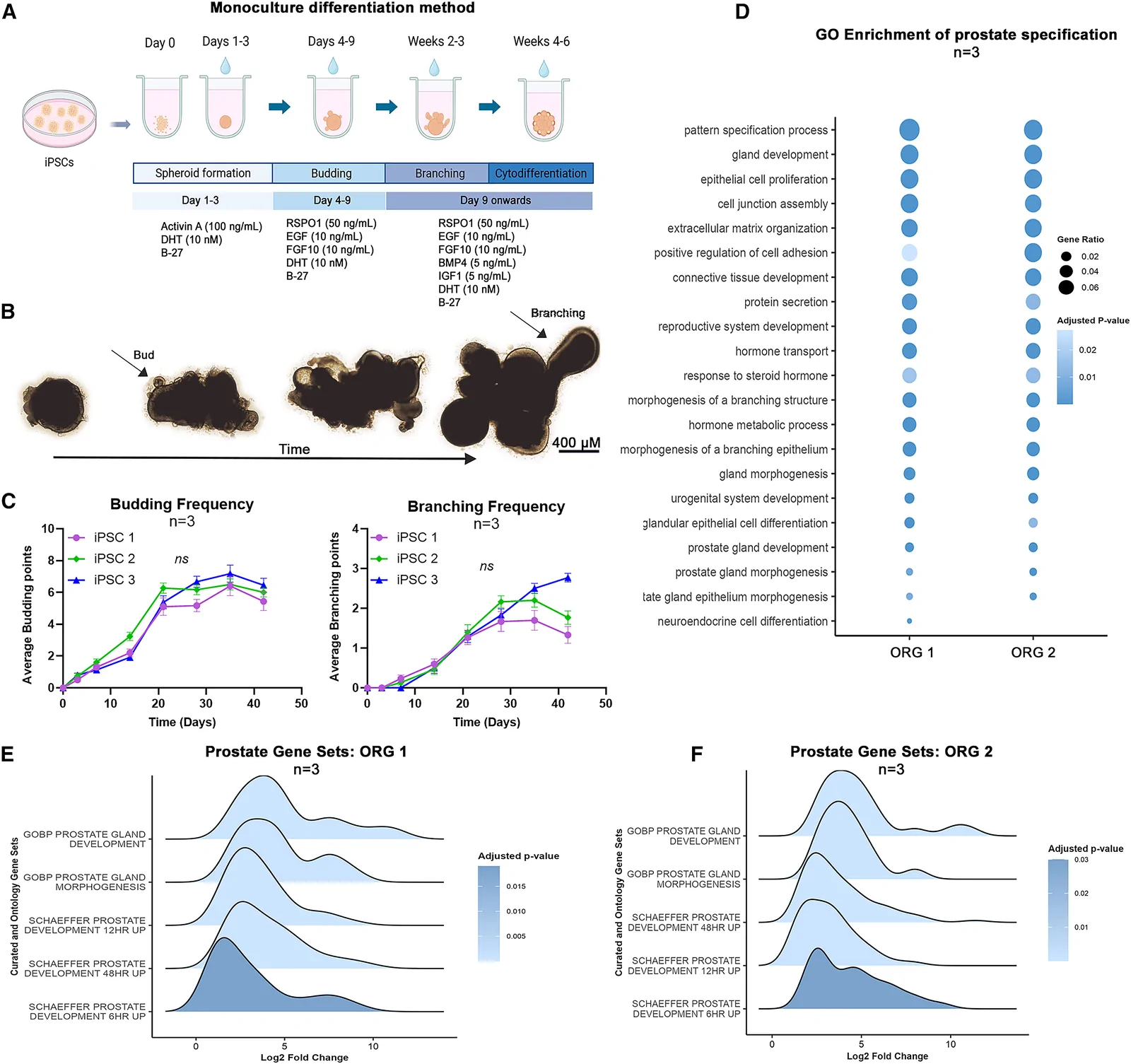
Human iPSC-derived prostate organoids self-organize and recapitulate key features of prostate development at week 6 (A) Schematic of the stepwise monoculture differentiation protocol from iPSCs, illustrating key stages of spheroid formation, budding, branching, and cytodifferentiation. (B) Representative bright-field images showing progressive organoid growth and morphogenesis from week 1 to week 6. Scale bars, 400 μm. (C) Quantification of budding and branching frequencies (*n* = 3) across three independent iPSC lines at week 6. Budding refers to the emergence of a single protrusion from the organoid surface, while branching involves further extension or bifurcation of these protrusions. A total of 90 organoids were assessed (30 per iPSC line: iPSC1, iPSC2, and iPSC3). These iPSC lines were derived from different donors and used as biological replicates to assess the consistency of developmental outcomes. (D) Gene Ontology (GO) enrichment analysis (*n* = 3) of bulk RNA-seq data from week 6 organoids derived from iPSC1 and iPSC2 reveals consistent biological processes related to prostate development. (E and F) Gene set enrichment analysis (GSEA) (*n* = 3) of week 6 organoids derived from iPSC1 (E) and iPSC2 (F) versus undifferentiated iPSCs shows significant upregulation of prostate development gene signatures. The top-enriched gene sets include human GO terms related to prostate gland development and morphogenesis. Additional enrichment of Schaeffer prostate development signatures, originally defined in a mouse model, highlights conserved androgen-responsive transcriptional programs recapitulated in the human organoids.

We generated three iPSC lines from healthy prostate tissues obtained from cystoprostatectomy surgery for bladder cancer, as previously described.^23^ All three cell lines were confirmed to be iPSCs by confirming their pluripotency and trilineage differentiation potential (Figure S1). To initiate prostate monoculture differentiation, iPSCs were aggregated into spheroids in low-attachment 96-well plates. On day 1, 3D spheroids were treated with Activin A to induce endodermal differentiation. By day 3, they expressed *SOX17* and *T* (*Brachyury*), confirming an early mesendodermal state (Figure S2A). At this point, spheroids were switched to prostate specification medium for 4 days, followed by prostate organoid medium for the remainder of the differentiation protocol (Figure 1A).

Throughout the 6-week differentiation period, organoids were live imaged to monitor morphological changes. Quantification of the budding and branching events at week 6 (Figures 1B and 1C) revealed consistent developmental outcomes across all three iPSC lines (*n* = 3; 30 organoids per iPSC line), demonstrating high reproducibility. No significant differences were observed among the lines. Most wells generated well-structured prostate organoids, with only a small fraction forming cystic, poorly organized structures. Overall, successful organoid formation ranged from 87.5% to 95% across independent differentiations (Figure S3A).

Two iPSCs and their derived organoids were characterized via transcriptomic analyses at week 6, and a clear transcriptional divergence between undifferentiated iPSCs and differentiating prostate organoids was confirmed using principal-component analysis (Figure S4A). Gene Ontology analysis (Figure 1D) showed the enrichment of biological processes associated with prostate specification. Gene set enrichment analysis (GSEA) (Figures 1E and 1F) further revealed strong upregulation of gene signatures linked to prostate gland differentiation. Notably, enrichment of the Schaeffer androgen responsive prostate development signature, originally defined in mouse, highlights the conserved nature of these developmental programs in our human organoids.^24^

### Acquisition of epithelial and stromal prostate lineages

**Figure 2:**
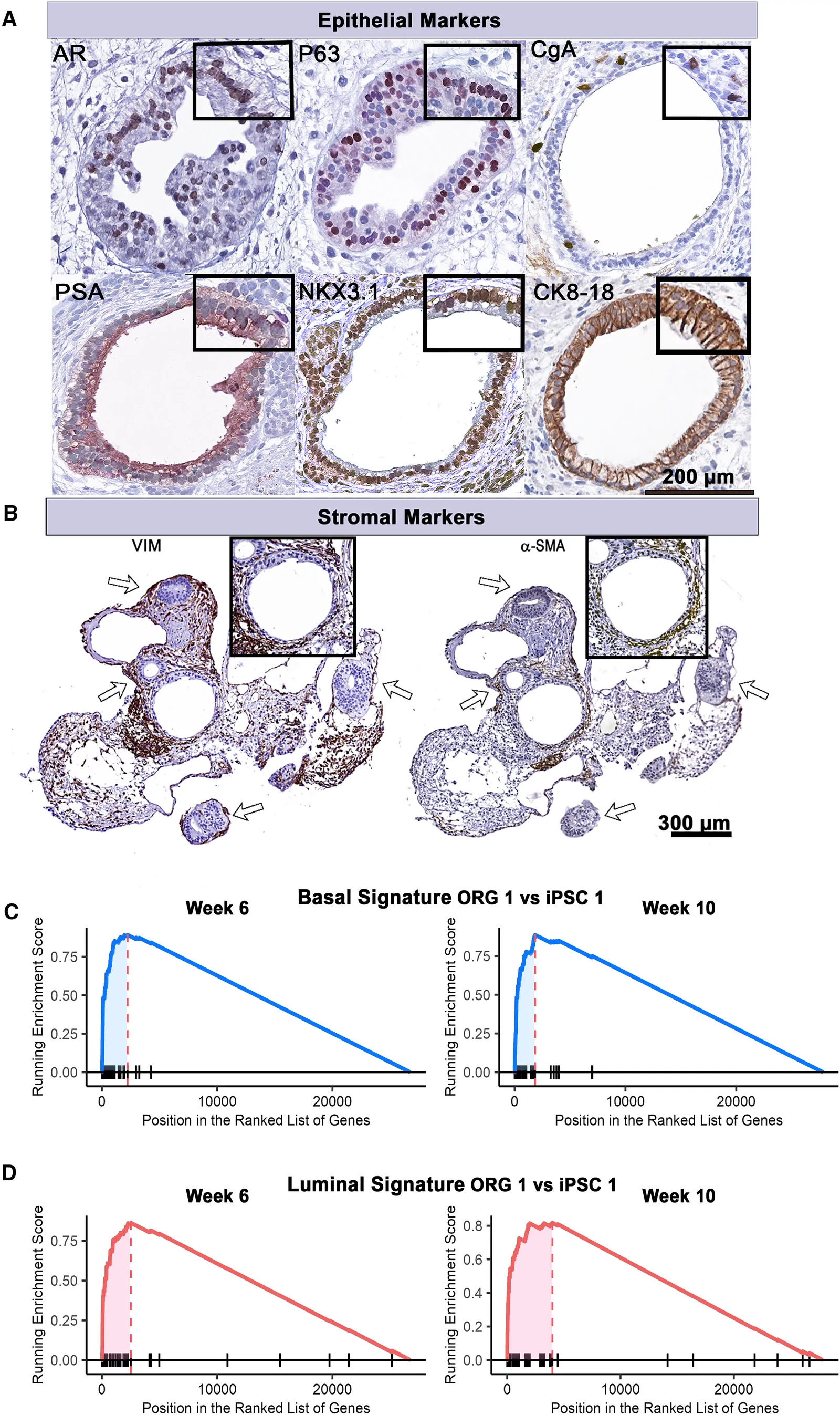
Prostate organoids recapitulate epithelial and stromal compartmentalization with progressive acquisition of tissue-specific gene signatures (A and B) Immunohistochemical staining of whole organoid sections reveals distinct epithelial regions expressing androgen receptor (AR), basal marker P63, neuroendocrine marker chromogranin A (CgA), prostate-specific antigen (PSA), NKX3-1, and cytokeratins 8 and 18 (CK8/18), supporting basal, luminal, and neuroendocrine differentiation. Immunostaining for stromal markers shows positive staining for vimentin (VIM) and α-smooth muscle actin (α-SMA). Black arrows indicate epithelial nests surrounded by stromal compartments. Scale bars, 200 and 300 μm. (C) Gene set enrichment analysis (GSEA) using basal signatures derived from primary adult prostate epithelial cells in organoids versus undifferentiated iPSCs at weeks 6 and 10, showing enrichment of basal genes. (D) GSEA using luminal signatures derived from primary adult prostate epithelial cells (*n* = 3) at weeks 6 and 10 shows progressive enrichment, consistent with luminal lineage specification over time.

Organoids from monoculture differentiation at week 6 were fixed, processed, and paraffin embedded for histological analysis. Bright-field imaging demonstrated progressive development from early spheroids at day 3 to budding at week 2, followed by increased branching and structural organization at weeks 4 and 6. This was accompanied by epithelial differentiation and luminal maturation, supported by increasing prostate-specific antigen (PSA) and CK8/18 expressions over time (Figures S3B and S3C). Immunohistochemistry (IHC) revealed clear epithelial and stromal compartmentalization, with epithelial nests expressing NKX3-1 and PSA and comprising luminal (CK8/18 and AR), neuroendocrine (chromogranin A), and basal (p63) cell populations. These epithelial structures were surrounded by a stromal compartment positive for vimentin and α-SMA, consistent with normal human prostate histology^25^^,^^26^ (Figures 2A and 2B).

Transcriptomic analysis revealed epithelial lineages consistent with prostate lineage specification. Basal signatures included *TP63* and *KRT5*, while luminal signatures encompassed *FOXA1*, *AGR2*, *CEACAM6*, *TFF3*, and *KRT19*. Neuroendocrine-associated transcripts such as *CHGA*, *SYP*, and *ENO2* were also detected (Figures S4C and S4D). Classical epithelial keratins CK8 (*KRT8*) and CK18 (*KRT18*) were abundantly expressed and readily detected by IHC, although their transcript levels varied between clones, with evidence of increased expression in ORG2 organoids. GSEA using basal and luminal signatures derived from primary adult prostate epithelial cells further supported these lineage identities, showing significant enrichment of basal epithelial programs at week 6 and enhanced acquisition of luminal signatures at week 10 (Figures 2C and 2D). This temporal pattern indicated a transition from basal-enriched to luminal-enriched states, mirroring the maturation trajectory described by Hepburn et al.,^23^ where iPSC-derived prostate organoids advance along the epithelial differentiation pathways during development.

Beyond the epithelial lineage, the expression of stromal genes, including *VIM*, *FAP*, *ACTA2*, *PDGFRB*, and *PDGFRA*, was maintained or increased during maturation, consistent with the histological identification of a well-developed stromal compartment surrounding the epithelial nests. Notably, we observed robust upregulation of extracellular matrix genes such as *LAMB1*, *ECM1*, *ECM2*, *COL22A1*, and *COL6A6*, highlighting the capacity of these organoids to self-organize and synthesize their own extracellular matrix under defined scaffold-free conditions.^27^^,^^28^ Together, these profiles reflect a multicompartmental architecture with epithelial, stromal, and extracellular-matrix-producing elements in defined domains (Figures S4B–S4D).

Originating from a common iPSC progenitor, this monoculture system is well suited for developmental studies and for modeling inherited conditions. It also enables the introduction of germline cancer-risk alleles, ensuring that both epithelial and stromal compartments share the same genetic context.

Together, these findings indicate that the organoid system recapitulates key features of prostate epithelial differentiation and tissue organization, consistent with established human prostate biology.

### Androgen supplementation is essential for epithelial differentiation

To assess the contribution of androgen signaling to prostate organoid monoculture differentiation, we cultured organoids with or without dihydrotestosterone (DHT) and assessed marker expression at weeks 4 and 6, using IHC.

**Figure 3:**
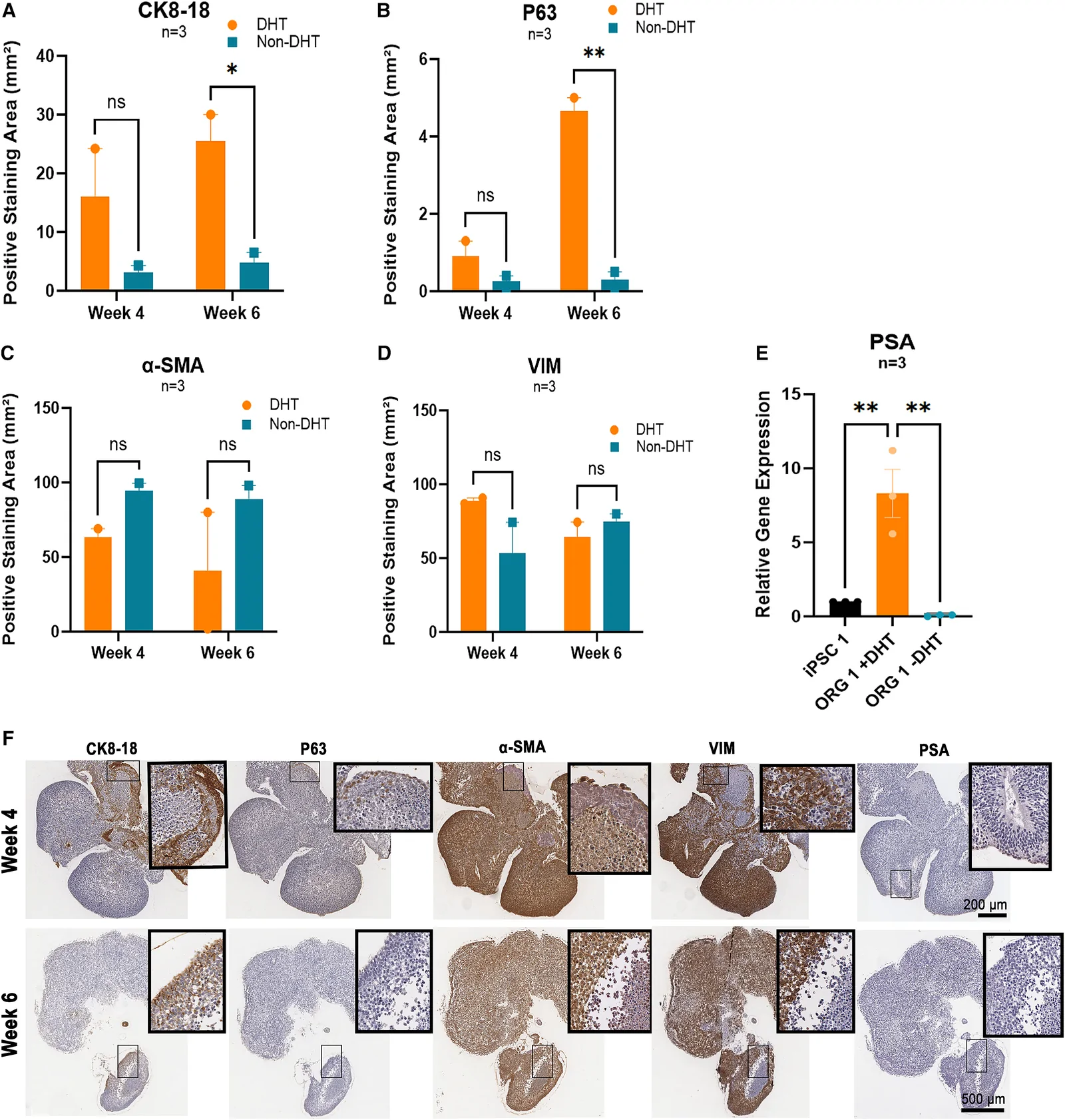
Androgen supplementation enhances epithelial differentiation in iPSC-derived prostate organoids (A and B) Bar plots showing positive staining area for the epithelial markers CK8/18 (A) and P63 (B) at weeks 4 and 6. DHT-treated organoids (orange bars) show increased staining over time, while androgen-deprived conditions (blue bars) show reduced epithelial marker expression. (C and D) Equivalent plots for the stromal markers α-SMA (C) and VIM (D) indicate that stromal marker expression is preserved or increased in the absence of DHT. (E) Quantification of *KLK3* (PSA) transcript expression by qPCR (*n* = 3) shows reduced levels in androgen-deprived conditions compared with DHT-treated organoids. iPSC1 is shown as the baseline control. Data are presented as mean ± SEM; ∗*p* < 0.05. (F) Representative immunohistochemistry images of whole organoid sections at weeks 4 and 6 cultured without DHT, stained for epithelial markers CK8/18, P63, and PSA, and the stromal markers α-SMA and VIM. Control images at week 6 are shown in Figures 2A and 2B. Scale bars, 200 and 500 μm.

Bar plots were generated from IHC images (*n* = 3) to quantify the marker-positive area. This analysis showed that organoids cultured with DHT exhibited increased expression of epithelial markers CK8/18 and p63 over time, while those in androgen-deprived conditions showed reduced expression of both markers (Figures 3A and 3B). In contrast, expression of the stromal markers α-SMA and vimentin was maintained or elevated in the absence of DHT, indicating that stromal differentiation does not depend on androgen signaling (Figures 3C and 3D).

Quantification of KLK3 (PSA) expression by qPCR (*n* = 3) showed a significant reduction under androgen-deprived conditions compared with DHT-treated organoids, with iPSC1 included as a baseline reference (Figure 3E; mean ± standard error of the mean [SEM], ∗*p* < 0.05). This reduction is consistent with impaired luminal maturation, although differences in the epithelial cell composition may also contribute. Representative IHC images (Figure 3F) support these observations. Organoids cultured without DHT displayed markedly lower staining for CK8/18, p63, and PSA, while α-SMA and vimentin expression remained detectable, consistent with previous reports that stromal markers are less affected by androgen withdrawal.^29^^,^^30^^,^^31^

### Co-culture strategy enables compartment-specific manipulation

**Figure 4:**
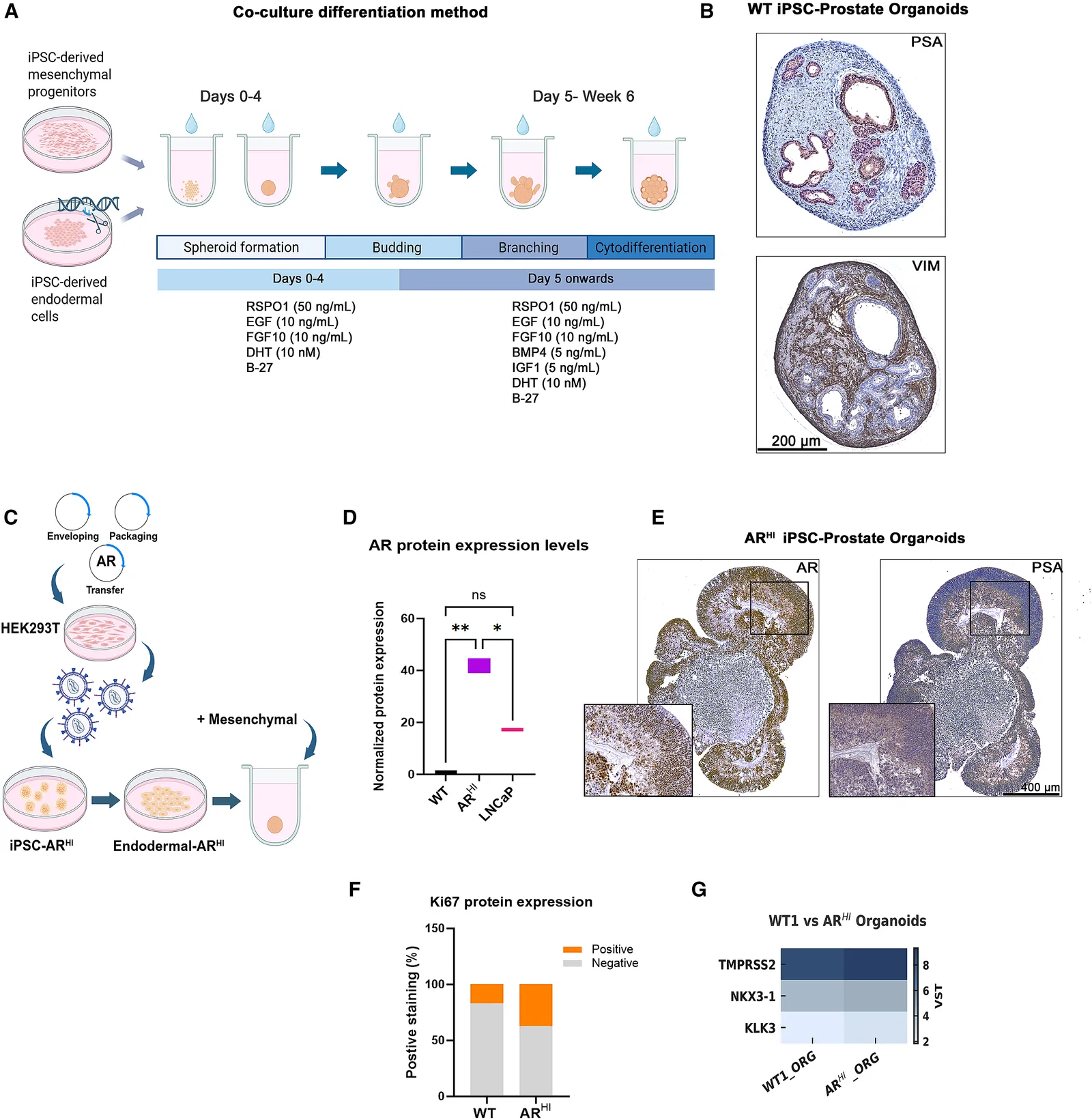
Co-culture model combining engineered endodermal cells and mesenchymal progenitors enables prostate organoid generation and epithelial compartment-specific genetic manipulation (A) Schematic of the co-culture protocol using an assembloid-like approach, in which iPSC-derived mesenchymal progenitors are combined with engineered iPSCs differentiated into endodermal cells. This system enables spatial compartmentalization of the stromal and epithelial lineages, supporting targeted manipulation of the epithelial compartment. Developmental stages, namely spheroid formation, budding, branching, and cytodifferentiation, are shown. (B) Immunohistochemistry of wild-type (WT) organoids shows PSA-positive epithelial regions and VIM-positive stromal regions. (C) Overview of the epithelial-specific manipulation strategy. The *AR* transgene was introduced into iPSCs, which were differentiated into endodermal cells (endodermal-*AR*ᴴᴵ) and co-cultured with mesenchymal progenitors to generate prostate organoids. (D) Quantification of AR protein levels by western blotting in iPSC-WT, iPSC-*AR*ᴴᴵ, and LNCaP cells, showing increased AR expression in iPSC-*AR*ᴴᴵ compared with WT controls. (E) Immunohistochemistry of *AR*ᴴᴵ organoids shows epithelial expression of AR and PSA, confirming epithelial targeting. Scale bars, 400 μm. (F) Ki67 staining reveals a higher proportion of proliferating cells in *AR*ᴴᴵ organoids compared with WT controls. (G) Heatmap of variance-stabilized (VST) expression values shows the induction of canonical AR targets (*TMPRSS2*, *NKX3-1*, and *KLK3*) in *AR*ᴴᴵ organoids.

While the monoculture system generates epithelial and stromal compartments from a common iPSC-derived progenitor, the co-culture system was designed to permit independent specification and manipulation of the epithelial and mesenchymal lineages prior to assembly. We next tested whether prostate organoids could be assembled by combining definitive endodermal progenitors (epithelial precursors) and mesenchymal progenitors (stromal precursors) using an assembloid-like method for organoid generation, enabling independent genetic manipulation of each compartment. This approach involved the directed differentiation of iPSCs into lineage-specific progenitors, followed by aggregation into spheroids and subsequent specification into prostate organoids (Figure 4A).

On day 3, gene expression analysis confirmed successful induction of definitive endoderm, as shown by the increased expression of SOX17 and FOXA2 in 2D iPSC cultures, together with downregulation of the pluripotency markers NANOG and OCT4. By day 21, mesenchymal progenitors showed high expression of NCAM1 and reduced levels of epithelial (EpCAM) and pluripotency markers (NANOG and OCT4). T (Brachyury) and MIXL1, transient markers associated with early mesoderm and mesendoderm specifications, were also downregulated, indicating progression toward a committed mesenchymal fate (Figure S2).

These data confirm that the epithelial and stromal progenitors are specified as distinct lineages prior to 3D assembly. IHC of co-cultured organoids at week 10 revealed clear compartmentalization, with PSA-positive epithelial regions and vimentin-positive stromal regions (Figure 4B), consistent with epithelial derivation from endodermal progenitors and stromal derivation from mesenchymal inputs.

As proof-of-concept for epithelial-specific genetic manipulation, we introduced an AR transgene into iPSCs prior to endoderm differentiation. Although AR is expressed in both epithelial and stromal compartments *in vivo*, here, AR overexpression was restricted to the endoderm-derived epithelial lineage to demonstrate compartment-specific manipulation. These engineered cells (endodermal-AR^HI^) were then co-cultured with unmodified mesenchymal progenitors to generate compartmentalized organoids (Figure 4C). Quantitative analysis showed significantly elevated AR protein levels in AR^HI^ iPSCs compared with wild-type control (Figure 4D).

IHC of AR^HI^ organoids confirmed robust AR and PSA expression restricted to the epithelial compartment, demonstrating successful targeting (Figure 4E). Ki67 staining further showed an increased proportion of proliferating cells in AR^HI^ organoids compared with wild-type controls, indicating that AR overexpression may enhance epithelial proliferation (Figure 4F). Transcriptomic profiles indicated elevated expression of canonical AR targets, including *KLK3*, *NKX3-1*, and *TMPRSS2*, in AR^HI^ organoids (Figure 4G). This co-culture system offers a platform that supports spatially organized prostate-like organoids and enables targeted manipulation of the epithelial lineage.

These two approaches provide complementary insights. Monoculture differentiation enables controlled modeling of prostate lineage specification from a common progenitor, whereas the assembloid approach introduces independently specified epithelial and mesenchymal compartments, allowing compartment-specific manipulation within a more complex epithelial-stromal context.

### Xeno-free human iPSC-derived prostate organoids display diverse epithelial and stromal identities at single-cell resolution

**Figure 5:**
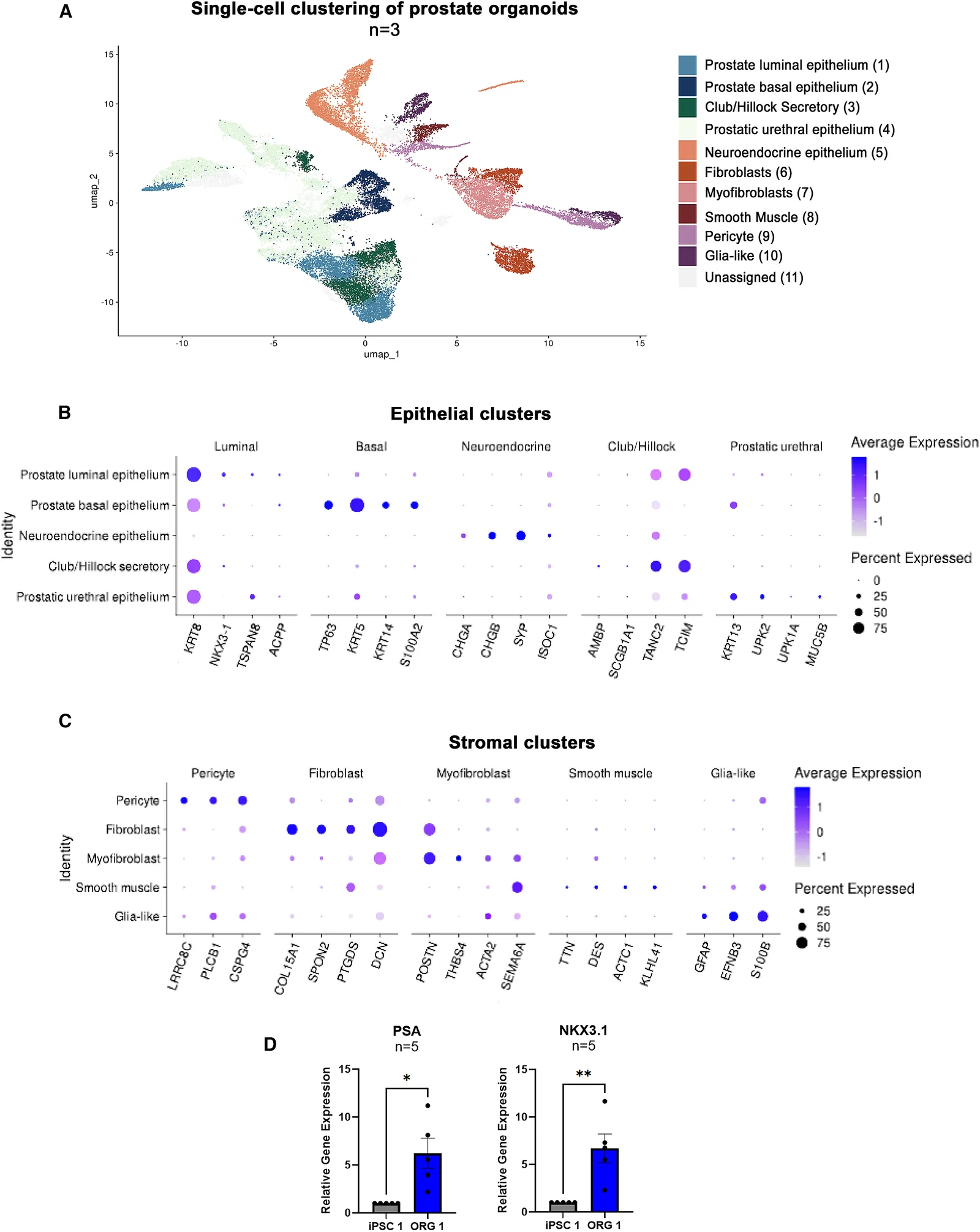
Single-cell transcriptomic profiling of wild-type iPSC-derived prostate organoids (A) Uniform manifold approximation and projection (UMAP) of week 10 wild-type (WT) organoids (*n* = 3) showing distinct epithelial and stromal clusters. Epithelial subtypes include prostate luminal, basal, club/hillock secretory, prostatic urethral, and neuroendocrine epithelium, while stromal lineages comprise fibroblasts, myofibroblasts, smooth muscle cells, pericytes, and glia-like cells. (B and C) Dot plots showing representative epithelial and stromal markers in week 10 organoids. Epithelial clusters: luminal: *KRT8*, *NKX3-1*, *TSPAN8*, and *ACPP* (androgen-responsive secretory epithelium); basal: *TP63*, *KRT5*, *KRT14*, and *S100A2* (structural/progenitor layer); neuroendocrine: *CHGA*, *CHGB*, *SYP*, and *ISOC1* (vesicle-associated hormone secretory cells resembling prostate neuroendocrine epithelium); club/hillock: *AMBP*, *SCGB1A1*, *TANC2*, and *TCIM* (regenerative/secretory subset); urethral-like: *KRT13*, *UPK2*, *UPK1A*, and *MUC5B* (transitional epithelium of the prostatic urethra). Stromal clusters: pericytes: *LRRC8C*, *PLCB1*, and *CSPG4* (perivascular/mural support); fibroblasts: *COL15A1*, *SPON1*, *PTGDS*, and *DCN* (ECM synthesis and paracrine signaling); myofibroblasts: *POSTN*, *THBS4*, *ACTC2*, and *SEMA6A* (matrix remodeling and contractility); smooth muscle: *TTN*, *DES*, *ACTC1*, and *KLHL41* (contractile cytoskeleton); glia-like: *GFAP*, *EFNB3*, and *S100B* (neural-associated stroma). (D) qPCR (*n* = 5) showing increased *KLK3* (PSA) and *NKX3-1* expressions in week 10 organoids (ORG1) compared with iPSCs (iPSC1); mean ± SEM; *p* < 0.05, *p* < 0.01.

Single-cell RNA sequencing of week 10 wild-type organoids generated using the modular epithelial-mesenchymal co-culture system (assembloid-like approach), representing the baseline co-culture model before epithelial-specific genetic manipulation, revealed epithelial and stromal diversity supporting the acquisition of prostate lineage identity (Figure 5A). The epithelial compartment comprised basal, luminal, club or hillock, neuroendocrine, and urethral-like populations, while the stromal compartment included smooth muscle, fibroblast, myofibroblast, pericyte, and glia-like cells.

Cluster annotations were validated using canonical lineage markers, consistent with known prostate epithelial identities. Luminal cells expressed KRT8, NKX3-1, TSPAN8, and ACPP, whereas basal epithelial cells expressed TP63, KRT5, KRT14, and S100A2. Neuroendocrine clusters were defined by CHGA, CHGB, SYP, and ISOC1, and the club or hillock subset expressed SCGB1A1 and AMBP, together with TANC2 and TCIM (Figure 5B).

The urethral-like epithelial population, characterized by the expression of KRT13, UPK2, and UPK1A, was consistently detected across organoids alongside basal, luminal, and club or hillock populations, consistent with the proximal origin of prostate epithelial lineages and the inclusion of urethral-associated epithelial states within the differentiation spectrum. Expression patterns across epithelial clusters further supported their transcriptional distinction (Figures S5A–S5D).

Stromal marker expression is shown in Figure 5C, including those of fibroblast genes (*COL15A1*, *SPON1*, *PTGDS*, and *DCN*), myofibroblast genes (*POSTN*, *THBS4*, *ACTA2*, and *SEMA6A*), smooth muscle markers (*TTN*, *DES*, *ACTC1*, and *KLHL41*), pericyte markers (*LRRC8C*, *PLCB1*, and *CSPG4*), and glia-like markers (*GFAP*, *EFNB3*, and *S100B*). These expression profiles correspond to the expected stromal phenotypes, with the myofibroblast cluster showing partial overlap between the fibroblast and smooth muscle signatures, consistent with a transitional role in matrix remodeling. Consistent with these identities, ligand-receptor analysis revealed predicted stromal-epithelial interactions, including the extracellular matrix-integrin and growth factor signaling pathways, supporting a functional role for stromal populations in epithelial organization (Figure S6A).

Orthogonal validation supported these identities. qPCR confirmed luminal differentiation by increased *KLK3* and *NKX3-1* expression (Figure 5D). Immunostaining identified desmin-positive stromal regions, *KRT13*-positive transitional epithelium, and chromogranin A (CgA)-positive neuroendocrine cells (Figure S5E). Together, these data demonstrated that xeno-free iPSC-derived prostate organoids recapitulate epithelial and stromal complexity and exhibit distinct lineage compartmentalization at single-cell resolution. Integration with published fetal and adult prostate single-cell datasets showed that the organoid epithelial populations exhibit transcriptional similarity to the adult luminal and basal compartments, while stromal populations show more variable correspondence (Figure S6C).

## Discussion

Organoid models have significantly advanced prostate research, providing tractable systems to study development, disease, and therapeutic responses. Tissue-derived models have enabled the expansion of benign and malignant epithelium, identification of progenitor populations, and generation of PDOs for drug testing.^12^^,^^14^^,^^32^^,^^33^ Despite their impact, these approaches have notable limitations, as they often lack a stromal component, are difficult to manipulate genetically after establishment, and do not easily recapitulate early developmental processes.^18^ Prolonged culture of primary tissue-derived organoids introduces selective pressures, and transcriptional drift affects the interpretation of results.^17^^,^^34^^,^^35^

To help address these limitations, we developed a defined, xeno-free and scaffold-free iPSC-based system that generates multilineage organoids with epithelial and stromal compartments. Our system captures key aspects of prostate lineage specification and tissue organization. The organoids show morphogenetic behaviors such as budding and branching, which are consistent with developmental milestones described *in vivo*.^36^

In a monoculture system, differentiation begins with the formation of a transient mesendodermal state marked by the co-expression of *SOX17* and *T (Brachyury)*. This bipotent intermediate is a recognized precursor to both mesoderm and endoderm lineages,^37^^,^^38^^,^^39^ providing a developmental foundation for generating multilineage organoids with epithelial-mesenchymal compartmentalization.^40^^,^^41^^,^^42^

As differentiation progresses, prostate organoids derived from single iPSC lines exhibit increasing expression of key epithelial markers, including AR, NKX3-1, PSA (*KLK3*), CK8/18, and p63. GSEA confirmed the activation of basal and luminal transcriptional programs, in agreement with previous observations.^23^ Although bulk RNA sequencing (RNA-seq) may not resolve rare or transitional subtypes, the presence of both basal and luminal signatures indicates successful epithelial specification. This setup is especially useful for modeling germline mutations, a growing application of iPSC-based systems in disease modeling and developmental biology.^17^^,^^43^

Our observations also align with the developmental stages previously described by Cunha and Baskin^44^ who showed that prostate bud initiation during the initial stages of development can occur in the absence of androgens, while branching and epithelial maturation require androgen signaling. Consistently, we found that organoids cultured without DHT exhibited reduced expression of epithelial markers such as CK8/18, p63, and PSA, while stromal markers like α-SMA and vimentin remained robust or elevated. This suggests that stromal differentiation may be less dependent on androgen signaling than epithelial development. This platform also provides an opportunity to investigate the timing and mechanisms of androgen and AR signaling during prostate epithelial differentiation in a controlled setting. Importantly, the observed epithelial differentiation, androgen responsiveness, and transcriptional alignment with published datasets are consistent with established features of prostate biology, supporting the validity of the model as a human *in vitro* system.

To enable lineage-specific manipulation, we developed a co-culture system in which epithelial and stromal compartments are specified independently. Genetic modification can be introduced prior to differentiation into definitive endoderm (*FOXA2*^*+*^ and *SOX17*^*+*^), which is subsequently combined with wild-type mesenchymal progenitors (*NCAM*^*+*^). This approach enables targeted gene editing within the epithelial lineage while preserving a physiologically relevant stromal environment. The resulting organoids displayed distinct epithelial (*PSA*^*+*^ and *AR*^*+*^) and stromal (*VIM*^*+*^ and *α-SMA*^*+*^) domains, consistent with organized prostate-like architecture. To demonstrate the utility of this system for disease modeling, an *AR* transgene was introduced into iPSCs prior to differentiation. The resulting organoids showed robust AR and PSA expression restricted to the epithelial compartment, while stromal cells remained unmodified. Increased Ki67 staining in AR^HI^ organoids is consistent with increased epithelial proliferation, a feature relevant to both normal prostate development and *AR*-driven tumorigenesis. Transcriptomic profiling revealed higher read counts for canonical *AR* target genes (*KLK3*, *NKX3-1*, and *TMPRSS2*) in AR^HI^ organoids. Collectively, these findings demonstrate that the co-culture system enables controlled, lineage-specific modeling of oncogenic drivers within a spatially organized epithelial-stromal environment.

Single-cell RNA sequencing of wild-type organoids revealed basal (*TP63*, *KRT5*, and *KRT14*), luminal (*NKX3-1*, *TSPAN8*, and *ACPP*), and neuroendocrine (*CHGA*, *CHGB*, and *SYP*) populations, together with club/hillock clusters representing secretory epithelial subsets. These populations collectively reflect the epithelial diversity of the developing prostate. As expected for probe-based single-cell approaches, transcripts with low abundance or unstable 3′ ends, such as *KLK3* (encoding PSA), showed reduced detection despite clear luminal identity confirmed by qPCR and immunostaining.^45^^,^^46^ The coexistence of progenitor and differentiated epithelial phenotypes suggests an actively remodeling epithelium, and the balance between these states can be modulated by the differentiation timing or inductive cues. This dynamic organization provides a framework to investigate developmental reactivation processes linked to prostate cancer. Integration with the reference datasets further supports epithelial lineage identity, with organoid epithelial populations showing transcriptional similarity to adult prostate cell states, while stromal populations display greater variability, consistent with a developing and context-dependent niche.

Stromal identities were supported by single-cell markers for smooth muscle (*ACTA1* and *DES*), fibroblast (*DCN* and *COL15A1*), pericyte (*PLCB1* and *CSPG4*), and glia-like populations (*S100B* and *EFNB3*), with clear module enrichment across stromal clusters. Desmin immunostaining further corroborated smooth muscle differentiation in the organoid stroma,^47^ aligning the transcriptomic calls with spatial protein expression. Although stromal subtypes partially overlapped in marker usage, their gene modules were distinct and consistent across replicates, supporting the robustness of lineage representation. Predicted ligand-receptor interactions further support a role for stromal populations in providing a signaling niche that contributes to epithelial organization and differentiation. Functional perturbation of these pathways represents a future direction enabled by this platform.

The single-cell landscape captured a fuller breadth of prostate lineage programs, including the prostatic urethra, that share a common embryonic development from the urogenital sinus epithelium.^36^^,^^48^^,^^49^ Specifically, a subset of epithelial cells expressing *KRT13*, *UPK2*, and *UPK1A* is consistent with the cells of the prostatic urethra and proximal ducts, as demonstrated by recent reports of whole human prostate single-cell characterization^26^^,^^50^^,^^51^ The interaction between these cells, along with club and hillock epithelial cell types, provide a model to explore their potential role in BPH.^26^ Importantly, genetic manipulation is introduced at the level of epithelial progenitor cells prior to differentiation, such that edits are propagated across epithelial lineages. Consequently, the presence of urethral-like epithelial cells does not preclude the use of the model to study epithelial genetic alterations.

This iPSC-derived system complements tissue-derived models by providing a reproducible, developmentally informed platform with matched epithelial and stromal organization. Recent Matrigel-free PDO studies have shown that eliminating animal-derived matrices preserves prostate cancer lineage fidelity and reduces transcriptional artifacts,^52^ underscoring the importance of defined, xeno-free conditions for translational relevance. Because the organoids generated here include transitional states and a urethral-like program, they provide a useful framework to investigate the processes relevant to BPH, including the reactivation of developmental pathways.^53^^,^^54^ The modular design, which allows lineage-specific genetic manipulation before aggregation, further enables mechanistic studies of how defined drivers and cues shape fate choices in a human system and support studies on differentiation, lineage specification, BPH, and malignant transformation. This platform also enables compartment-specific genetic perturbation, providing an opportunity to investigate stromal-epithelial signaling in a human context. Future studies could leverage this system to examine AR function across epithelial and mesenchymal compartments, as well as key regulators such as NKX3-1.

### Limitations of the study

This report presents a defined protocol and an initial experimental series spanning two differentiation time points—week 6 and week 10. Probe-based single-cell RNA sequencing was conducted at week 10 to characterize lineage diversity once the epithelial and stromal compartments had become clearly distinguishable, providing a representative snapshot of prostate-like tissue organization, rather than a full developmental trajectory. Earlier changes were assessed by bulk transcriptomics and immunostaining, which complement the single-cell dataset. Cell composition reflects the patterning cues and culture duration used here and can be further refined in future experiments to favor specific epithelial outcomes. The current system does not yet include immune or vascular components; so, those interactions are not yet represented.

While this study establishes and validates the platform, systematic functional benchmarking and targeted perturbation studies are important next steps to further define its capacity to model specific aspects of prostate biology.

## Resource availability

### Lead contact

Requests for further information, resources, and reagents should be directed to and will be fulfilled by the lead contact, Adriana Buskin (adriana.buskin@ncl.ac.uk).

### Materials availability

This study did not generate new unique reagents.

### Data and code availability

• Bulk and single-cell RNA sequencing datasets generated during this study can be found at the following link: https://doi.org/10.5281/zenodo.20427357.
• This paper does not report original code.
• Any additional information required to reanalyze the data reported in this paper is available from the lead contact upon request.

## Acknowledgments

This work was primarily supported by the 10.13039/501100000849National Centre for the Replacement, Refinement and Reduction of Animals in Research (NC3Rs; project NC/W002396/1, Training Fellowship awarded to A.B.). We also acknowledge additional support from 10.13039/501100000771Prostate Cancer UK (project TLD-CAF23-007) and the 10.13039/100000892Prostate Cancer Foundation (project 18CHAL11). Acquisition of the Live-Cell Analysis System was enabled by an MRC equipment grant with the support of Dr. Kelly Coffey. Financial support to M.L. from the IMI2 StemBANCC initiative is also gratefully acknowledged.

## Author contributions

Conceptualization, A.B. and R. Heer; methodology, A.B.; investigation, L.J.W., L.W., R. Howarth, S.S., J.L., A.C.H., R.N., R. Hussain, J.C., and A.B.; formal analysis, N.S., M.T., and A.B.; data curation, N.S., and M.T.; visualization: N.S., M.T., and A.B.; resources, A.C.H., L.G., and M.L.; supervision, E.S., C.N.R., and A.B.; funding acquisition, A.B.; writing – original draft, A.B.; writing – review & editing, N.S., M.T., Q.L., B.S., S.W.H., D.W.S., R. Heer, and A.B.

## Declaration of interests

The authors declare no competing interests.

## STAR★Methods

### Key resources table

**Table:** 

REAGENT or RESOURCE	SOURCE	IDENTIFIER
**Antibodies**		

OCT4	Merck Millipore	Cat# MABD76; RRID: AB_10919170
SOX2	Merck Millipore	Cat# MAB4343; RRID: AB_827493
NANOG-Alexa Fluor 647	Cell Signaling Technology	Cat# 5448 S; RRID: AB_10694485
TRA-1-60-FITC	Merck Millipore	Cat# FCMAB115F; RRID: AB_10563509
AR	BD Biosciences	Cat# 554225; RRID: AB_395316
NKX3-1	Origene UltraMAB	Cat# UM800088; RRID: AB_2629197
CK8-18	Abcam	Cat# ab17139; RRID: AB_443679
CK18	Proteintech	Cat# 10830-1-AP; RRID: AB_2133164
P63	Leica Novocastra	Cat# NCL-L-p63; RRID: AB_3720967
Vimentin (D21H3)	Cell Signaling Technology	Cat# 5741; RRID: AB_10695459
α-SMA	Abcam	Cat# ab7817; RRID: AB_262054
α-SMA	Abcam	Cat# ab5694; RRID: AB_2223021
PSA	Santa Cruz Biotechnology	Cat# sc-7316; RRID: AB_2279058
Chromogranin A	Abcam	Cat# ab52983; RRID: AB_2081119
Desmin	Proteintech	Cat# 16520-1-AP; RRID: AB_2292918
KRT13	Proteintech	Cat# 10164-2-AP; RRID: AB_2134679
Collagen IV	Abcam	Cat# ab6586; RRID: AB_305584

**Biological samples**		

Human prostrate specimens	Freeman Hospital	N/A

**Chemicals, peptides, and recombinant proteins**		

ROCK inhibitor Y-27632	STEMCELL Technologies	Cat# 72304
DMEM-F12 medium	Thermo Fisher Scientific	Cat# 11330
FBS	Thermo Fisher Scientific	Cat# 10270
RPMI 1640	Thermo Fisher Scientific	Cat# 11875093
GlutaMAX	Thermo Fisher Scientific	Cat# 35050061
Activin A	STEMCELL Technologies	Cat# 78034
CHIR99021	Tocris	Cat# 4423
RSPO1	Thermo Fisher Scientific	Cat# A42586
EGF	Thermo Fisher Scientific	Cat# PHG0315
FGF10	PeproTech	Cat# 100-26
BMP4	Thermo Fisher Scientific	Cat# PHC9534
IGF1	Thermo Fisher Scientific	Cat# 78142
B-27 Supplement	Thermo Fisher Scientific	Cat# 17504044
B-27 Supplement minus Insulin	Thermo Fisher Scientific	Cat# A1895601
mTeSR1 medium	STEMCELL Technologies	Cat# 85850
Vitronectin	Thermo Fisher Scientific	Cat# A14700
Versene	Thermo Fisher Scientific	Cat# 15040066
Accutase	STEMCELL Technologies	Cat# 07920

**Critical commercial assays**		

CytoTune™-iPS 2.0 Sendai Reprogramming Kit	Thermo Fisher Scientific	Cat# A16517
STEMdiff™ Mesenchymal Progenitor Kit	STEMCELL Technologies	Cat# 05240

**Deposited data**		

Bulk and single-cell RNA-seq datasets	Zenodo	https://doi.org/10.5281/zenodo.20427357

**Experimental models: Cell lines**		

Human iPSC line 1	This paper	iPSC1
Human iPSC line 2	This paper	iPSC2
Human iPSC line 3	This paper	iPSC3
LNCaP cells	ATCC	Cat# CRL-1740

**Oligonucleotides**		

Primer sequences	This paper	See Table S1

**Recombinant DNA**		

MYC_pLX307 vector	Addgene	Cat# 98363

**Software and algorithms**		

Cell Ranger	10*x* Genomics	v8.0.0
Seurat	Hao et al.^55^	v4.4.0
DESeq2	Love et al.^56^	v1.42.1
Salmon	Patro et al.^57^	v1.10.1
DoubletFinder	McGinnis et al.^58^	v2.0.4
Harmony	Korsunsky et al.	v1.2.4

**Other**		

Ultra-low attachment 96-well plates	Corning costar	Cat# 7007

### Experimental model and study participant details

#### Patient material

All surgical specimens were collected in accordance with local ethical and regulatory guidelines, with written informed consent from patients (Newcastle REC 2003/11 and Human Tissue Authority License 12,534, Freeman Hospital, Newcastle upon Tyne, United Kingdom). The anonymized samples were labeled iPSC 1, iPSC 2, and iPSC 3, corresponding to patients aged 82, 81, and 67 years at the time of surgery.

#### iPSC generation

Enriched cultures of 1 × 10^5^ primary human prostate stromal cells were seeded in 12-well plates and transduced using the CytoTune-iPS 2.0 Sendai virus reprogramming kit (Thermo Fisher Scientific, A16517), delivering OCT4, SOX2, KLF4, and c-MYC, according to the manufacturer’s instructions. Emerging induced pluripotent stem cell (iPSC) colonies were first established on an inactivated primary mouse embryonic fibroblast feeder layer and subsequently transitioned to feeder-free culture conditions as described below.

Established iPSC lines were maintained in mTeSR1 medium (StemCell Technologies, 85850) on vitronectin (Thermo Fisher Scientific, A14700). Pluripotency was confirmed by expression of canonical markers, including OCT4, NANOG, and SOX2, assessed by immunofluorescence and qPCR. iPSC lines were further validated by karyotype analysis and tri-lineage differentiation potential.^23^

### Method details

#### iPSC culture

Human iPSCs were cultured in six-well plates coated with vitronectin (Thermo Fisher Scientific, A14700) using mTeSR1 medium (StemCell Technologies, 85850). The culture medium was replaced daily. Cells were allowed to grow for 4–5 days, before either passaging or initiating differentiation. For passaging, cells were treated with Versene solution (Thermo Fisher Scientific, 15040066) at 37°C for 3–5 min, and then transferred to fresh Vitronectin-coated plates at a ratio of 1:3 to 1:6. All cultures were maintained in a humidified environment at 37°C with 5% CO_2_.

#### iPSC characterization

Pluripotency markers in iPSC colonies were detected using immunocytochemistry and flow cytometry. Colonies were fixed in 4% paraformaldehyde (Sigma-Aldrich, 47608), permeabilized with 0.25% Triton X-100 (Sigma-Aldrich, T8787), and blocked with 10% FBS and 1% bovine serum albumin (Sigma-Aldrich, A3311) before staining with anti-human SOX2 (Merck Millipore, MAB4343, 1:200) and anti-human OCT4 (Merck Millipore, MABD76, 1:200). Secondary staining was performed using Alexa Fluor 647 goat anti-mouse (Thermo Fisher Scientific, A21235, 1:400), followed by DAPI counterstaining at 1 μg/mL (Thermo Fisher Scientific, D1306), and imaged with a Leica DM6 microscope. For flow cytometry, iPSCs were dissociated with Accutase (StemCell Technologies, 07920), stained with TRA-1-60-FITC (Merck Millipore, FCMAB115F, 1:60) and NANOG-Alexa Fluor 647 (Cell Signaling Technology, 5448 S, 1:150), and analyzed using a Fortessa *X*20 system. For *in vitro* three-germ-layer differentiation, iPSCs were treated with Dispase (StemCell Technologies, 07923), cultured in differentiation media containing DMEM-F12 (Thermo Fisher Scientific, 11330), 20% FBS (Thermo Fisher Scientific, 10270), and stained for germ-layer markers using the antibodies AFP (Thermo Fisher Scientific, MA5-14666), TUBB3 (BioLegend, 801201), and α-SMA (Abcam, ab5694). Negative controls used only secondary antibodies.

#### qPCR

Reverse transcription was performed on 1 μg of RNA using MMLV reverse transcriptase (Promega, M1701) following the manufacturer’s instructions. Quantitative PCR was performed using the PowerTrack Master Mix (ThermoFisher, A46109) according to manufacturer’s guidance. Expression of markers (Table S1) was normalized to *RPL13A*.

#### iPSC engineering

The full-length androgen receptor (ARFL) cDNA was cloned into the MYC_pLX307 vector (Addgene, 98363) using T4 ligase (Thermo Fisher Scientific, IVGN2104), after removing the MYC sequence with the restriction enzymes NheI and SpeI (New England Biolabs, R3131S and R3133S). Bacterial transformation was carried out in Stbl3 chemically competent *E*. *coli* (Thermo Fisher Scientific, C737303), and plasmid purification was performed using the Invitrogen miniprep kit (Thermo Fisher Scientific, K210011). The presence of ARFL in the plasmid was confirmed through Sanger sequencing and diagnostic restriction digestion. The ARFL_pLX307 plasmid was packaged into lentivirus and resuspended in culture media following ultracentrifugation. iPSCs were transduced one day post-passage with 250 μL of viral particle solution for 48 h. Cells carrying the plasmid were selected using blasticidin (1 μg/mL), followed by clonal selection to ensure that all cells contained the AR construct.

#### Organoid generation

Organoids were generated under defined, xeno-free conditions without the use of exogenous extracellular matrix components. No Matrigel, collagen, or other scaffold materials were used at any stage. Instead, both monoculture and co-culture organoids were formed as free-floating three-dimensional aggregates in ultra-low attachment plates, allowing self-organization in suspension.

#### Monoculture

DE Spheroid Formation (days 0–3): Cells were first washed with PBS and incubated with 1 mL of Accutase Reagent (StemCell Technologies, 07920) for 3 min at 37°C to facilitate detachment. After incubation, 1 mL of mTeSR medium (StemCell Technologies, 85850) was added, and the cells were gently pipetted 1–3 times to generate a single-cell suspension. The suspension was transferred to a 15 mL conical tube and centrifuged at 300 × g for 5 min. Following centrifugation, the supernatant was discarded, and cells were resuspended in 1 mL of mTeSR medium per well collected. Viable cell concentration was determined using Trypan Blue (Thermo Fisher Scientific, 15250061) and a hemocytometer. The suspension was then adjusted to 1,000 cells per well in 100 μL of mTeSR medium supplemented with 10 μM ROCK inhibitor (StemCell Technologies, 72304). Cells were plated in ultra-low attachment 96-well plates (Corning Costar, 7007) and immediately centrifuged at 250 × g for 10 min at 20°C to encourage uniform cell settling. Plates were incubated at 37°C with 5% CO_2_ and 95% humidity. After 24 h, daily media changes were performed for 3 days using 100 μL of differentiation medium consisting of RPMI 1640 (Thermo Fisher Scientific, 11875093) supplemented with 1% Penicillin-Streptomycin (Thermo Fisher Scientific, 15140122), 1% GlutaMAX (Thermo Fisher Scientific, 35050061), 100 ng/mL Activin A (StemCell Technologies, 78034), 1× B-27, and 10 nM DHT.

Prostate Organoid Specification (Days 4–8): From days 4–8, prostate specification treatment was initiated by adding 100 μL of the following medium to each well daily: RPMI 1640 (Thermo Fisher Scientific, 11875093) supplemented with 1% Pen-Strep (Thermo Fisher Scientific, 15140122), 1% GlutaMAX (Thermo Fisher Scientific, 35050061), 50 ng/mL RSPO1 (Thermo Fisher Scientific, A42586), 10 ng/mL EGF (Thermo Fisher Scientific, PHG0315), 10 ng/mL FGF10 (Thermo Fisher Scientific, 100–26), 1× B27 (Thermo Fisher Scientific, 17504044), and 10 nM DHT.

Prostate Organoid Differentiation (Day 9 onward): Starting from day 9, prostate organoid differentiation treatment was initiated, with 100 μL per well added every other day. The treatment medium consisted of Advanced DMEM (Thermo Fisher Scientific, 12491015) with 1% Pen-Strep and 1% GlutaMAX, 50 ng/mL RSPO1, 10 ng/mL EGF, 10 ng/mL FGF10, 5 ng/mL BMP4 (Thermo Fisher Scientific, PHC9534), 5 ng/mL IGF1 (Thermo Fisher Scientific, 78142), 10 mM HEPES (Thermo Fisher Scientific, 15630056), 1*X* B27, 10 nM DHT, and 1 μL/mL Amphotericin B (Thermo Fisher Scientific, 15290018). Organoids were harvested at weeks 6 and 10 for further analysis.

#### Co-culture

For co-culture organoid generation, iPSC-derived definitive endoderm cells and mesenchymal progenitors were prepared separately under defined conditions as described below. Following lineage specification, the two populations were dissociated using Accutase (StemCell Technologies, 07920), centrifuged at 300 × g, and counted using Trypan Blue (Thermo Fisher Scientific, 15250061). The two cell types were combined at a 3:1 ratio (mesenchymal: endodermal) to a final density of 1,000 cells per well in 100 μL of media (as described in Step 2 of the monoculture protocol). Cells were seeded into ultra-low attachment 96-well plates, (Corning Costar, 7007) and immediately centrifuged at 250 × g for 10 min to encourage aggregation.

Definitive endoderm: iPSCs were dissociated into single cells using Gentle Cell Dissociation Reagent (StemCell Technologies, 07174) following the manufacturer’s instructions. A total of 5 × 10^5^ cells were seeded into Vitronectin-coated 6-well plates in mTeSR1 medium, (StemCell Technologies, 85850) supplemented with 10 μM ROCK inhibitor (StemCell Technologies, 72304). After 24 h, the medium was replaced with RPMI 1640 (Thermo Fisher Scientific, 11875093) containing 100 ng/mL human recombinant Activin A (StemCell Technologies, 78034) and 2.5 μM CHIR99021 (Tocris, 4423), without additional supplements. On Day 2, cells were washed once with PBS and cultured in RPMI 1640 supplemented with 100 ng/mL Activin A and B-27 minus insulin (Thermo Fisher Scientific, A1895601; 1:50 dilution). On Day 3, the medium was changed to RPMI 1640 containing 100 ng/mL Activin A and B-27 complete (Thermo Fisher Scientific, 17504044; 1:50 dilution). Definitive endoderm cells were harvested after 72 h for downstream use.

Mesenchymal progenitors: iPSCs were differentiated into mesenchymal progenitors over 21 days using the STEMdiff Mesenchymal Progenitor Kit (StemCell Technologies, 05240), following the manufacturer’s instructions. The protocol included three key stages: passaging iPSCs, induction of early mesodermal progenitors, and maturation into mesenchymal progenitor cells. Each stage involved coating culture plates, preparing single-cell suspensions, performing medium changes, and monitoring confluency and viability using Trypan Blue (Thermo Fisher Scientific, 15250061).

#### Real-time morphological analysis

Plates were placed in the Incucyte Live-Cell Analysis System and scanned at 4X magnification, capturing one image per well. The phase contrast/Brightfield channels were selected, with a spheroid growth scan type and a scan interval of every 6 h. Morphological changes were counted independently on days 3, 7, 14, 21, 28, 35, and 42. This assessment focused on two key features: the number of buds, defined as round structures emerging from the organoids, and branching, which refers to the expansion of buds or the connection of multiple buds together.

#### Histological analysis

##### Fixation, processing, and paraffin embedding of organoids

Organoids were harvested at weeks 6 or 10, washed in PBS, and fixed in 10% neutral buffered formalin (NBF) for 1 h at room temperature. Following fixation, samples were washed again with PBS, and Histogel (Thermo Fisher Scientific or equivalent) was melted in Eppendorf tubes using a dry heat block at 100°C for 5 min, then mixed with organoids and transferred into embedding molds. Molds were left at 4°C overnight to solidify. The following day, solidified Histogel plugs were transferred into tissue cassettes and placed in 70% ethanol for storage or immediate processing. Samples were processed through a graded alcohol and xylene series using a Leica tissue processor, then embedded in paraffin using a standard embedding station. Paraffin blocks were sectioned at 4–5 μm thickness using a microtome, and sections were mounted onto charged glass slides for downstream histological or immunohistochemical analysis.

##### Immunohistochemistry protocol for FFPE tissue slides

Formalin-fixed, paraffin-embedded (FFPE) tissue slides were deparaffinized in xylene and rehydrated through a graded ethanol series. Antigen retrieval was performed using heat-induced epitope retrieval in citrate buffer with a pressure-based decloaking chamber, heated to 121°C for 20 min. Slides were then cooled and rinsed under running water. Endogenous peroxidase activity was blocked using 3% hydrogen peroxide, and nonspecific binding was minimized by incubating the slides with blocking serum. Primary antibodies against AR, NKX3-1, CK8/18, CK18, p63, vimentin, α-SMA, PSA, chromogranin A, desmin, KRT13, and collagen IV were used for immunohistochemistry and immunofluorescence analyses as indicated. Detailed reagent information is provided in the Key Resources Table. Detection was achieved using DAB staining. Slides were counterstained with hematoxylin, dehydrated through ethanol, cleared in xylene, and mounted with DPX. Slides were scanned using the Aperio system for digital image capture and analysis.

### Quantification and statistical analysis

Statistical analyses were performed using GraphPad Prism and R software as indicated in the relevant sections. Data are presented as mean ± standard deviation (SD) or standard error of the mean (SEM), as specified in the figure legends. Pairwise comparisons were performed using two-sided Welch t-tests unless otherwise indicated. Differential gene expression analysis for bulk RNA-seq was performed using DESeq2 with significance thresholds of adjusted *p*-value <0.01 and absolute log2 fold change >1.

#### Gene expression analysis

##### Bulk RNA-seq analysis

RNA libraries were prepared using the NEBNext Ultra II RNA Library Prep Kit for Illumina (New England Biolabs) and sequenced on an Illumina NovaSeq platform (2 × 150 bp paired end), generating approximately 20 million reads per sample. Raw data quality was assessed using FASTQC v0.12.1,^59^ and adapter trimming was performed using Trim Galore v0.6.10.^60^ Transcript quantification was carried out using Salmon v1.10.1,^57^ with GENCODE Release 46 (GRCh38.p14) as the reference and the GRCh38 primary genome assembly used as a decoy to improve mapping accuracy. Transcript-level estimates were imported into R v4.3.2 using tximport v1.30.0^61^ from Bioconductor v3.18^62^ and summarized to gene-level counts. Transcript and gene identifiers were based on Ensembl release 111.^63^

Differential gene expression analysis was performed using DESeq2 v1.42.1,^56^ with genes considered significant at adjusted *p*-value (padj) < 0.01 and absolute log2 fold change >1. Principal Component Analysis (PCA) was conducted on variance-stabilized transformed (VST) data using the top 500 most variable genes. Volcano plots were generated from DESeq2 output to visualize the magnitude and significance of differential expression.

Gene Ontology (GO) enrichment was performed on differentially expressed genes (DEGs) using clusterProfiler v4.10.1,^64^ targeting Biological Process (GO BP) terms with *p*-value <0.05 and q-value <0.2 (Benjamini-Hochberg correction). Gene Set Enrichment Analysis (GSEA) was also conducted on ranked DEGs using prostate-relevant hallmark, curated, and ontology gene sets from msigdbr v7.5.1,^65^ with adjusted *p*-value <0.05.

Bulk RNA-seq datasets of sorted basal and luminal prostate cells from primary prostate epithelial tissue^66^ were used to derive reference signatures. The top 50 DEGs (padj <0.01) were selected to create curated gene sets. These were used in GSEA to assess basal and luminal enrichment across organoid samples.

For visualization, VST-normalized expression of epithelial, stromal, neuroendocrine, and extracellular matrix markers was averaged across replicates, compiled into matrices, and plotted as heatmaps using the pheatmap R package v1.0.12.^67^

##### Single-cell RNA-seq analysis

Single-cell libraries were generated using the 10*x* Genomics Fixed RNA Profiling v1 chemistry with the Human Transcriptome Probe Set v1.0.1, with sequencing performed by the Genomics Core Facility, Newcastle University. Reads were aligned to the GRCh38-2020-A reference transcriptome using Cell Ranger v8.0.0^68^ processed by the Bioinformatics Support Unit.

Gene expression matrices were imported into Seurat v4.4.0^55^ for downstream analysis. Cells were filtered to exclude those with fewer than 600 UMIs, fewer than 400 detected genes, a gene/UMI log10 ratio below 0.80, or mitochondrial content exceeding 10%. Genes expressed in fewer than 200 cells were excluded. Visualizations including histograms, violin plots, and scatterplots were used to guide quality control.

Each replicate was independently processed through the standard Seurat pipeline, including normalization, variable feature identification, scaling, and clustering. Doublets were detected and removed using DoubletFinder v2.0.4.^58^ After quality filtering, replicate Seurat objects were merged, and cell cycle effects were regressed out using the default gene sets in Seurat.

Dimensionality reduction was performed using Principal Component Analysis (PCA), followed by nearest neighbor identification and clustering. UMAP was used for visualization. Clusters were manually annotated using known prostate cell markers, and top markers within each cluster were identified based on a log2 fold change threshold of 0.25. The top 25 genes per cluster were used to refine annotations.

Integration of organoid, fetal^69^^,^^70^ and adult prostate datasets^26^ was completed in Seurat using representative prostate cell types. The three datasets were normalised using sctransform (v.0.4.3) and integrated with harmony (v1.2.4).

## Supplemental information

Document S1. Figures S1–S6 and Table S1

Document S2. Article plus supplemental information
