# Supplementary material for: A xeno-free human iPSC-derived prostate organoid platform for multilineage differentiation and genetic manipulation
Source: Cell Rep Methods. 2026 Jul 29;6(9):101538. doi: 10.1016/j.crmeth.2026.101538 (PMC13615492; doi:10.1016/j.crmeth.2026.101538)
Supplement: Supplementary file 1 [file mmc1.pdf]

Cell Reports Methods, Volume *6*

Supplemental information

A xeno-free human iPSC-derived prostate organoid platform for multilineage differentiation and genetic manipulation

Neha Shaikh, Matthew Teasdale, Lewis J. Walker, Laura Wilson, Rachel Howarth, Sara Saleem, Jamie Logsdon, Anastasia C. Hepburn, Ryan Nelson, Qiuyu Lian, Aleyda C. Elizondo, Rafiqul Hussain, Jonathan Coxhead, Luke Gaughan, Majlinda Lako, Emma Scott, Craig N. Robson, Ben Simons, Simon W. Hayward, Douglas W. Strand, Rakesh Heer, and Adriana Buskin

SUPPLEMENTARY INFORMATION

Xeno-free human iPSC-derived prostate organoid platform for multilineage differentiation and genetic manipulation

A

Sample	Age (biopsy/surgery)	Diagnosis
WT1	82 yrs	Benign
WT2	81 yrs	Benign
WT3	67 yrs	Benign

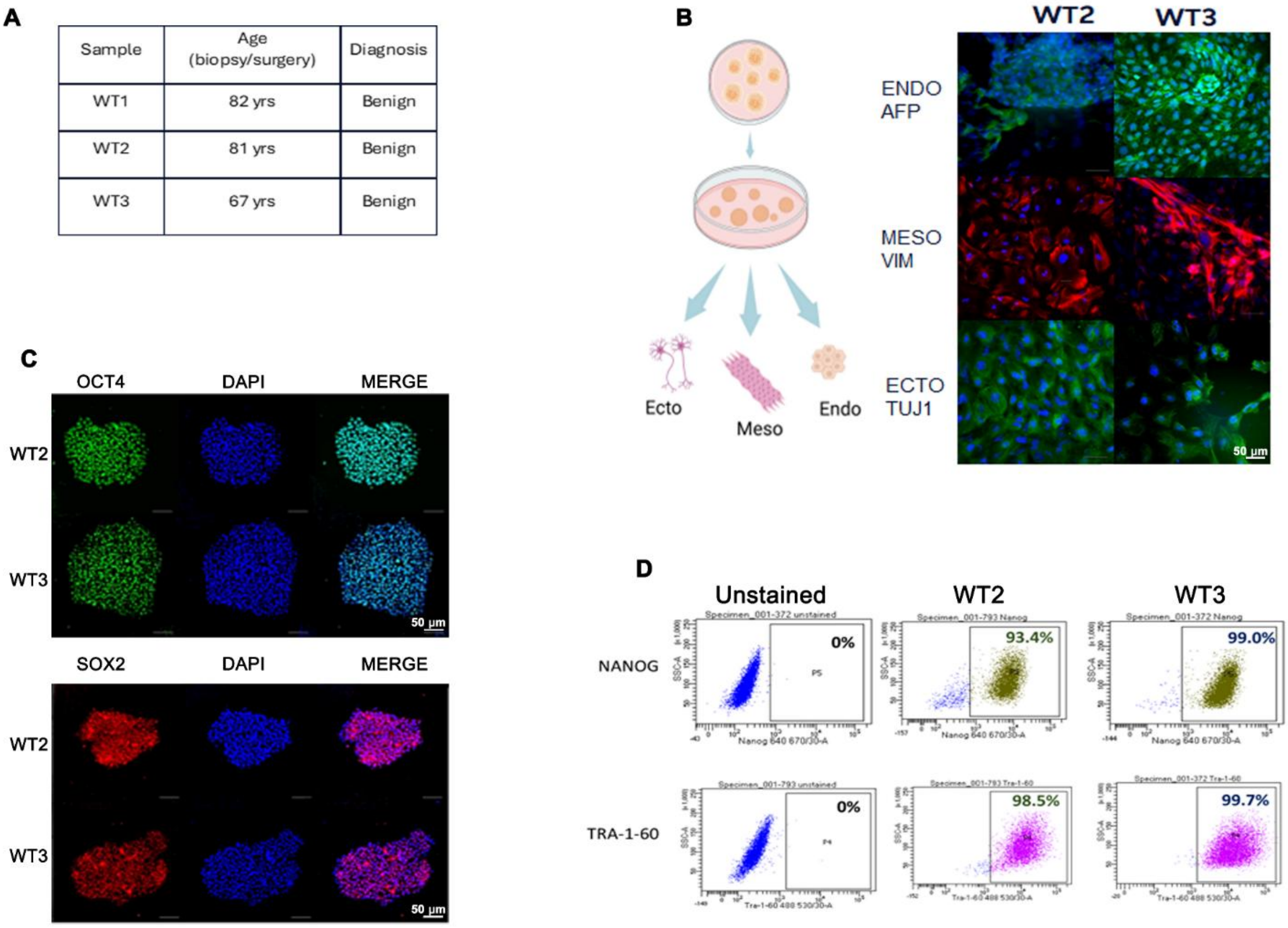

Supplementary Figure 1. Generation and characterization of iPSCs used for prostate differentiation. (A) Overview of the three iPSC lines used in this study. WT1 data were previously published in Hepburn et al. (23). (B) Trilineage differentiation assay demonstrating that iPSCs can generate endoderm, mesoderm, and ectoderm derivatives. (C) Immunofluorescence staining showing expression of pluripotency markers OCT4 and SOX2. (D) Flow cytometry analysis confirming expression of NANOG and TRA-1-60, supporting the pluripotent status of the iPSCs. Related to Figure 1.

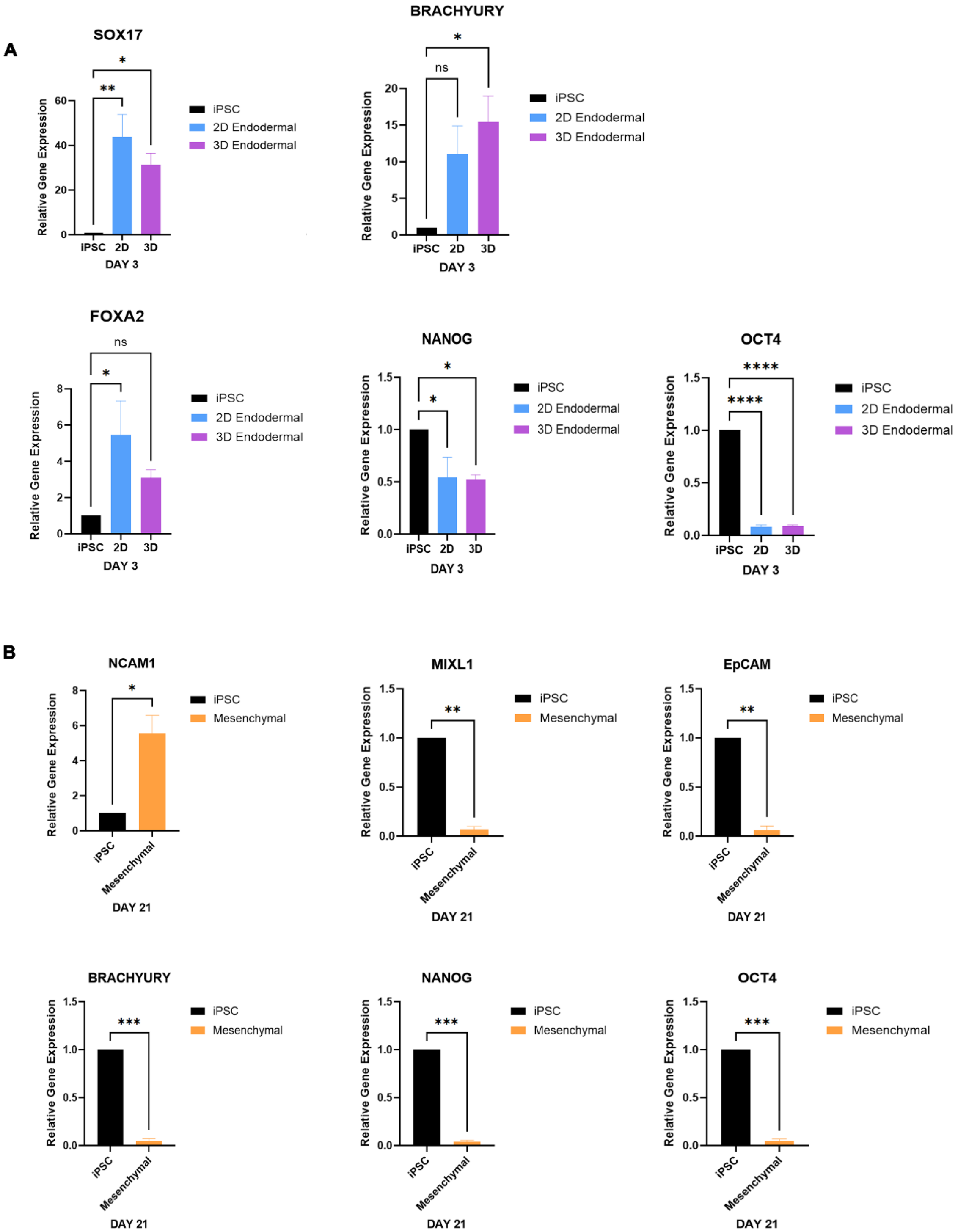

Supplementary Figure 2. Validation of endodermal and mesenchymal progenitor differentiation from iPSCs.

(A) Expression of markers *SOX17*, *T* (Brachyury), *FOXA2*, *NANOG* and *OCT4* in 3D and 2D cultures via qPCR. Expression of *SOX17* and *T* (Brachyury) in 3D spheroids suggests the presence of a mesendodermal intermediate. Downregulation of *NANOG* and *OCT4* indicates loss of iPSC identity after differentiation. n = 4 biological replicates per condition. (B) By day 21, differentiation to mesenchymal progenitors is supported by upregulation of *NCAM1* and loss of epithelial and pluripotency markers, with reduced *EPCAM*, *NANOG*, *OCT4*, and *T* (Brachyury). *MIXL1*, a transient early mesoderm marker, is downregulated, consistent with progression beyond early mesoderm to a committed mesenchymal fate. n=3 biological replicates per condition. Bars represent mean ± SD of relative gene expression quantified by $2^{-\Delta\Delta Ct}$, normalized to *RPL13A* and calibrated to iPSC = 1. Pairwise comparisons were performed using two-sided Welch *t*-tests. Significance: ns $p \geq 0.05$, * $p < 0.05$, ** $p < 0.01$, *** $p < 0.001$, **** $p < 0.0001$. Related to figures 1, 2 and 4.

A

iPSC line	Wells seeded	Glandular organoids	Cystic organoids	Failed wells (%)	Number of independent differentiations
iPSC1	32	28	4	12.5%	3
iPSC2	60	57	3	5%	3
iPSC3	30	27	3	10.0%	3

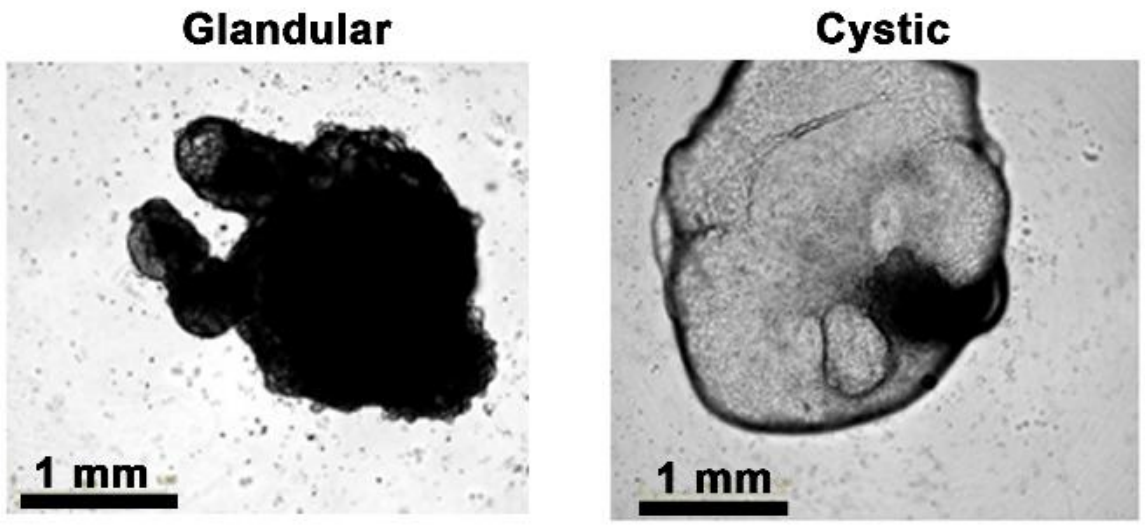

B

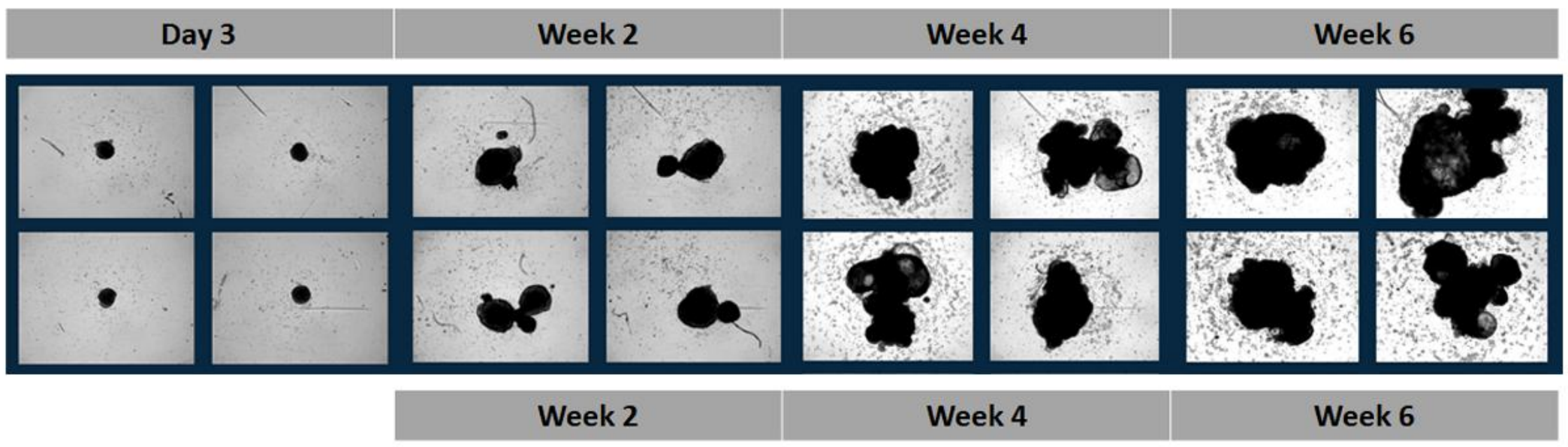

C

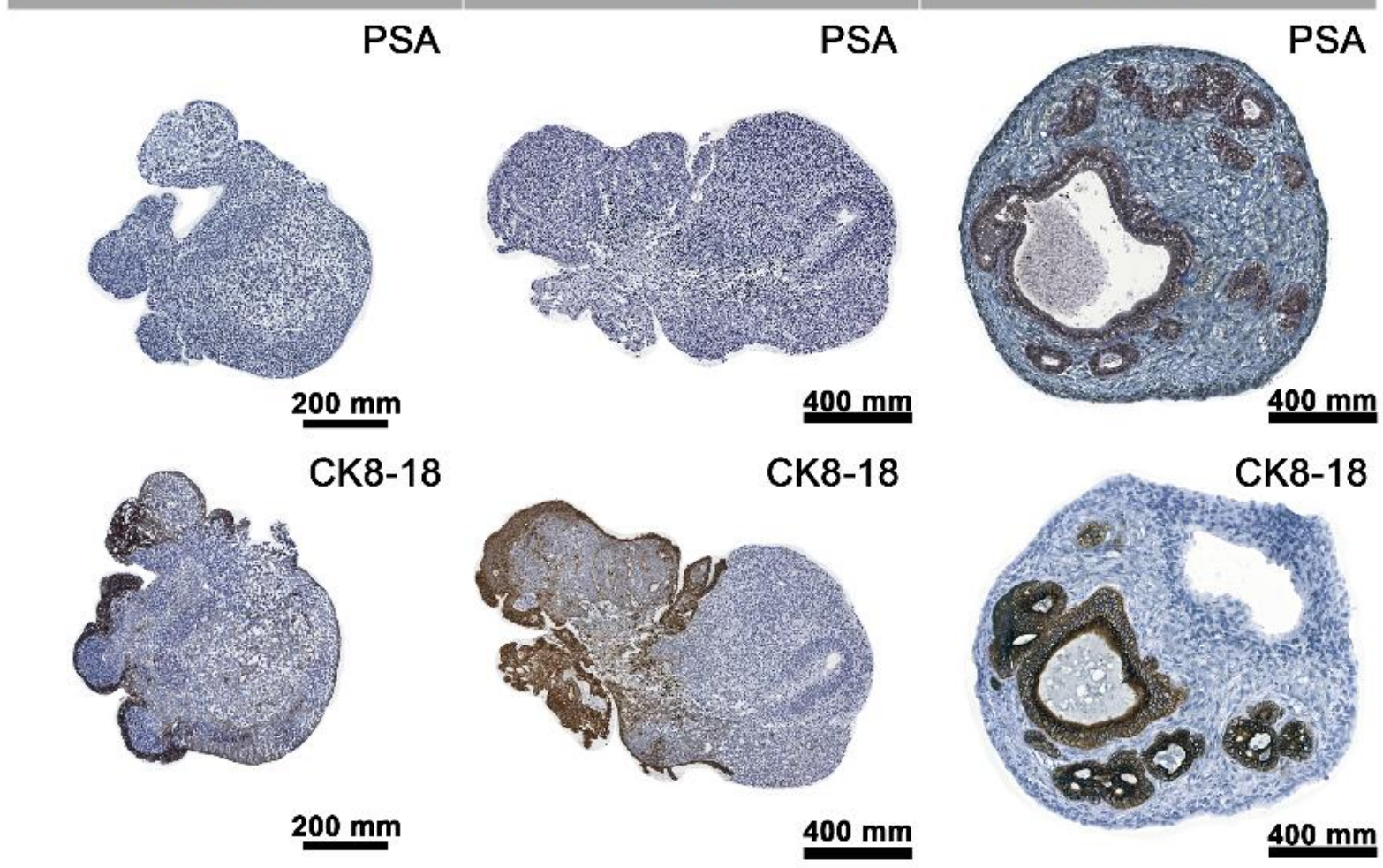

Supplementary Figure 3. Efficient and reproducible generation of glandular prostate organoids with progressive epithelial differentiation.

(A) Quantification of organoid outcomes across three independent iPSC lines, including wells seeded, glandular and cystic organoids formed, failed wells, and number of independent differentiations. Representative brightfield images of glandular and cystic organoids are shown. (B) Representative brightfield images showing organoid development spanning early spheroid formation (day 3), onset of budding (week 2), and later branching and structural organization (weeks 4 and 6). (C) Immunohistochemistry of organoid sections at weeks 2, 4, and 6 showing expression of PSA and CK8-18, consistent with progressive epithelial differentiation and luminal maturation. Scale bars as indicated. Related to figures 1, 2, 3 and 4.

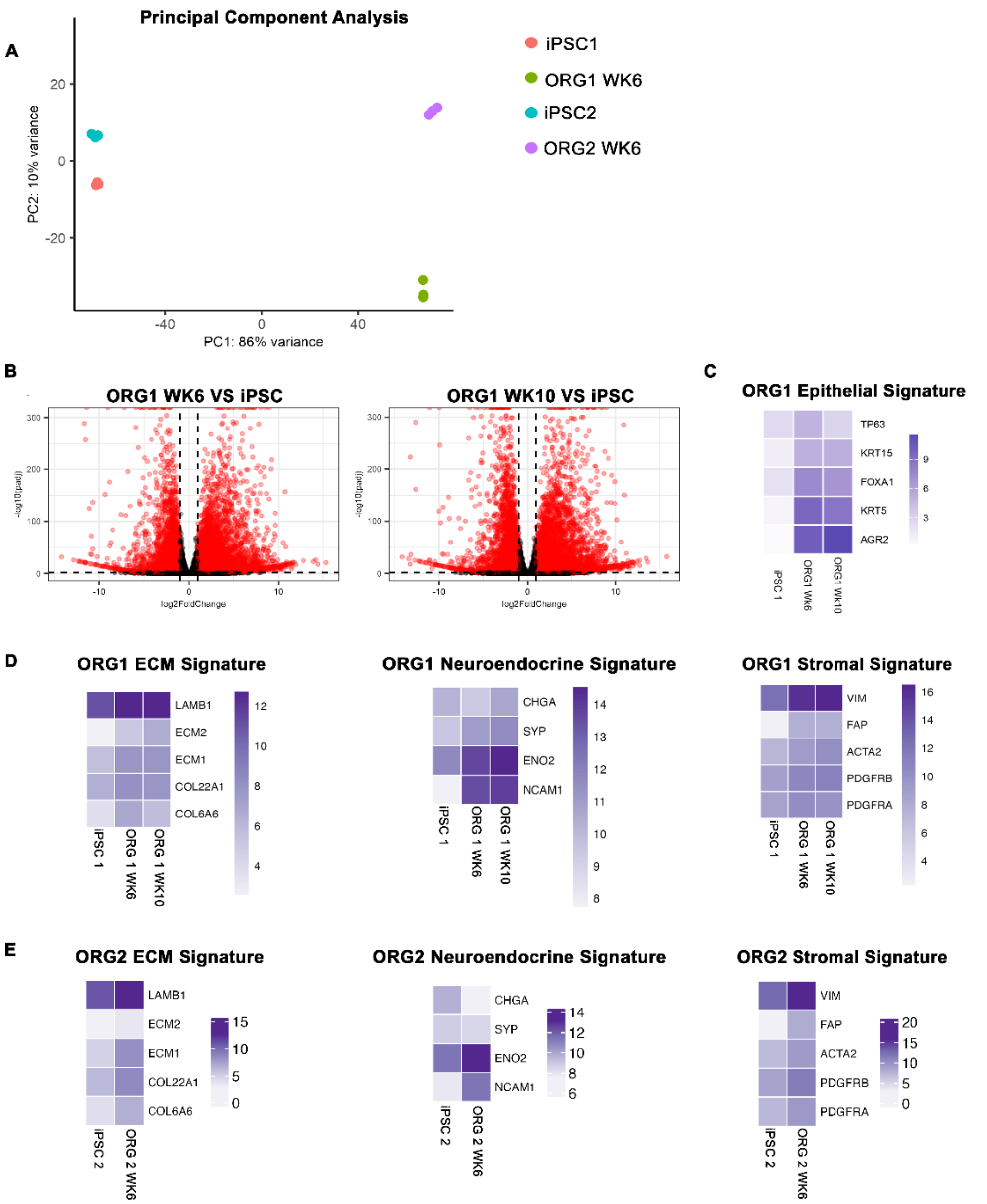

Supplementary Figure 4. Bulk RNA-seq reveals transcriptional changes and pathway enrichment associated with prostate organoid differentiation.
(A) Principal component analysis (PCA) shows transcriptional divergence between undifferentiated iPSCs and organoids at week 6, indicating distinct gene expression profiles following differentiation. (B) Volcano plots showing differentially expressed genes in organoids (ORG1) at weeks 6 and 10 compared with iPSCs, highlighting dynamic transcriptional changes during differentiation. (C) Heatmap of epithelial gene signatures in ORG1 organoids, week 6 and week 10 timepoints. (D) Heatmaps of selected gene signatures in ORG1 organoids, including extracellular matrix (ECM) genes (*LAMB1*, *ECM1*, *ECM2*, *COL22A1*, *COL6A6*), neuroendocrine markers (*CHGA*, *SYP*, *ENO2*, *NCAM1*), and stromal genes (*VIM*, *FAP*, *ACTA2*, *PDGFRB*, *PDGFRA*) across iPSC, week 6, and week 10 timepoints. (E) Equivalent ECM, neuroendocrine, and stromal signatures observed in ORG2 organoids at week 6 support reproducibility of lineage specification across biological replicates. Related to figures 1 and 2.

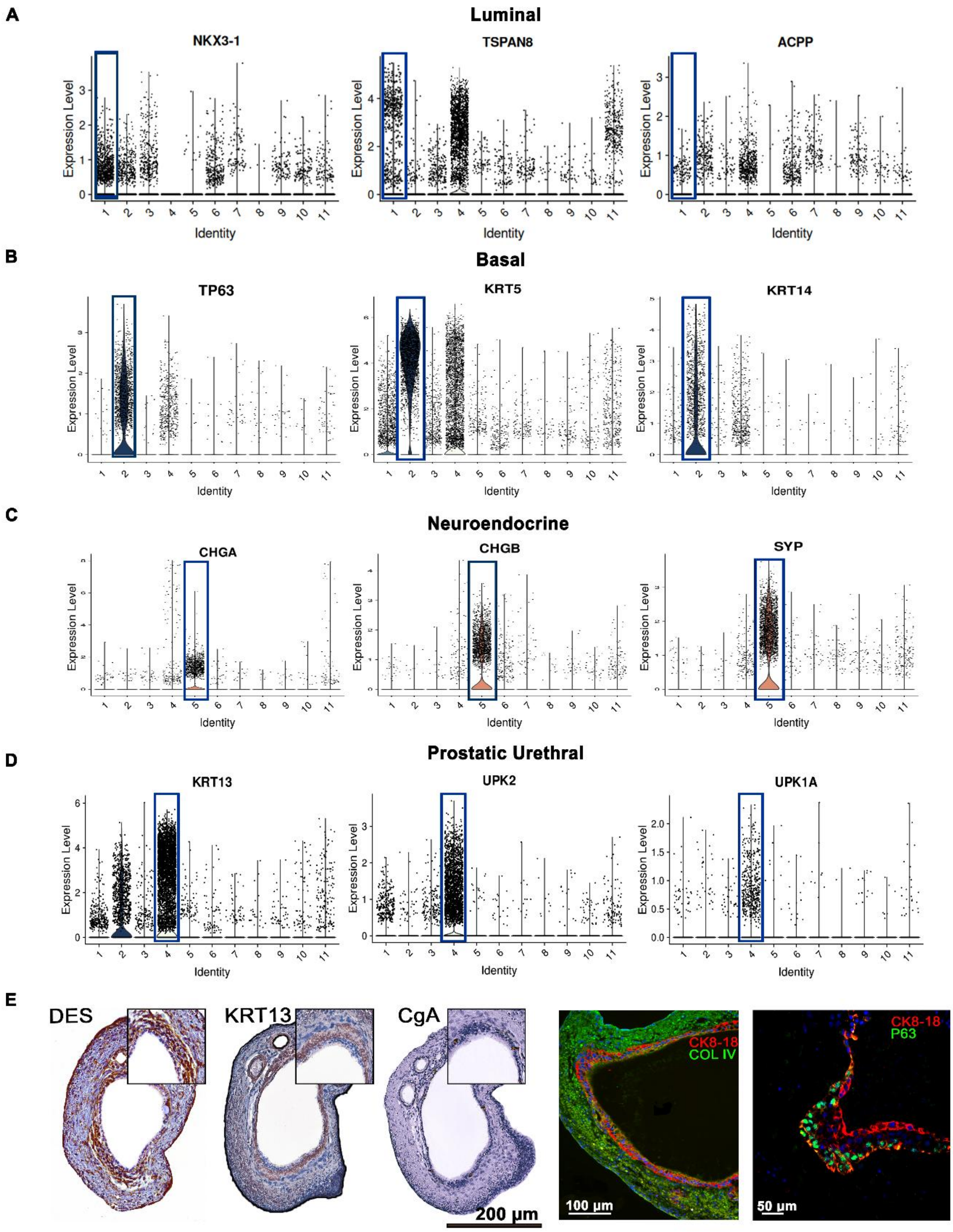

Supplementary Figure 5. Validation of prostate lineage acquisition in WT iPSC-derived organoids.

(A) Violin plots of luminal markers *NKX3-1*, *TSPAN8*, and *ACPP*, showing expression in the luminal cluster and detectable levels in other epithelial populations. (B) Violin plots of basal markers *TP63*, *KRT5*, and *KRT14*, showing expression in the basal cluster. (C) Violin plots of neuroendocrine markers *CHGA*, *CHGB*, and *SYP*, showing expression in the neuroendocrine cluster. (D) Violin plots of prostatic urethral cell markers *KRT13, UPK2*, and *UPK1A*, showing expression in the prostatic urethral epithelium cluster. (E) Immunohistochemistry for desmin (stromal), keratin 13 (urethral epithelium), and chromogranin A (neuroendocrine) showing spatial organization of prostate-relevant lineages. Immunofluorescence images for CK8-18 and collagen IV, CK8-18 and p63. **Cluster identities:** (1) Prostate luminal epithelium, (2) Prostate basal epithelium, (3) Club/Hillock secretory, (4) Prostatic urethral epithelium, (5) Neuroendocrine epithelium, (6) Fibroblast, (7) Myofibroblast, (8) Smooth muscle, (9) Pericyte, (10) Glia-like, (11) Unassigned. Scale bars as indicated. Related to figures 4 and 5.

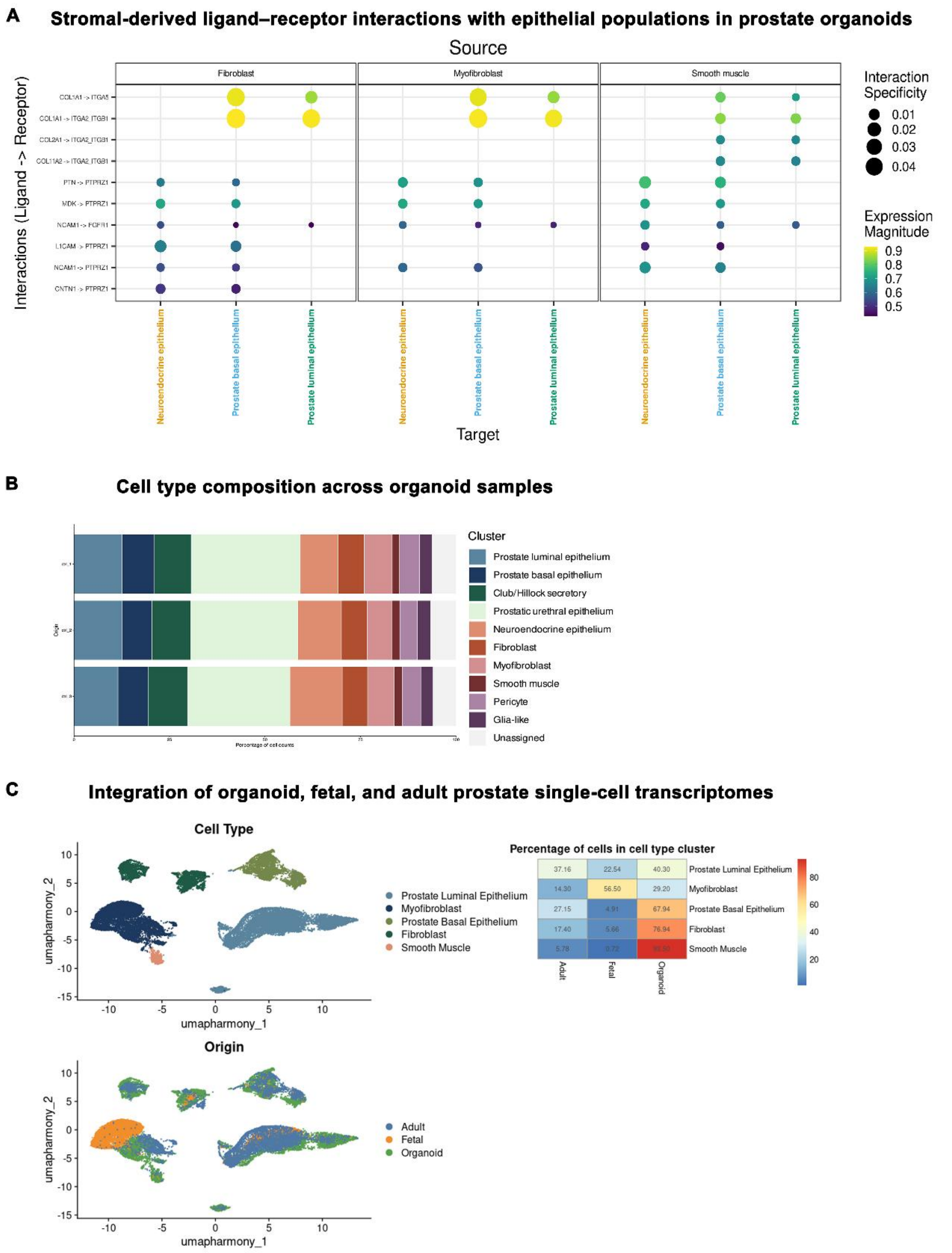

Supplementary Figure 6. Stromal–epithelial interactions and integration of iPSC-derived prostate organoids with fetal and adult prostate datasets.

(A) Dot plot showing predicted ligand–receptor interactions between stromal populations (fibroblasts, myofibroblasts, and smooth muscle) and epithelial cell types (luminal, basal, and neuroendocrine), inferred from single-cell RNA-seq data. (B) Stacked bar plots showing the relative proportions of epithelial and stromal cell populations across organoid samples. (C) UMAP projection of integrated single-cell transcriptomic data from organoid-derived, fetal[69,70] and adult prostate tissues[26], showing overlap of epithelial and stromal populations across datasets (cell type annotation is from this study). Related to figures 4 and 5.

Name	Sequence Forward	Sequence Reverse
PSA	CCTCACAGCTGCCCACTGCA	GATGAAACAGGCTGTGCCG
RPL13A	CCTGGAGGAGAAGAGGAAAGAGA	TTGAGGACCTCTGTGTATTTGTCAA
AR	GAAGTCCACGCTCACCATGTG	TACCAGCTCACCAAGCTCCTG
NKX3-1	CAGATAAGACCCCAAGTGC	CAGAGCCAGAGCCAGAG
FOXA2	TCCGACTGGAGCAGCTACTATG	CCACGTACGACGACGACATGTTC
SOX17	CGCACGGAATTTGAACAGTA	GGATCAGGGACCTGTCACAC
BRACHYURY	TGCTGCAATCCCATGACA	CGTTGCTCACAGACCACA
NANOG	CCAAATTCTCCTGCCAGTGAC	CACGTGGTTTCCAAACAAGAAA
OCT4	GCAAGCCCTCATTTCACCAGGCC	AGGATCAACCCAGCCCGGCT
NCAM1	AACAAAGCATGATGGGTGAA	GTCTGTGGTGTTGGAAATGC
MIXL1	TTTTCTCCCCTCTTCCAGGTA	CAGCTGGGGCTTCAGACATT
EPCAM	GGCTGCCAAATGTTTGGTGAT	TAAAGAGCCCGCTCTCATCG

Table S1. Primer sequences used in this study. Related to STAR methods.
